# Charge, Aspect Ratio, and Plant Species Affect Uptake Efficiency and Translocation of Polymeric Agrochemical Nanocarriers

**DOI:** 10.1021/acs.est.3c01154

**Published:** 2023-05-25

**Authors:** Yilin Zhang, Michael R. Martinez, Hui Sun, Mingkang Sun, Rongguan Yin, Jiajun Yan, Benedetto Marelli, Juan Pablo Giraldo, Krzysztof Matyjaszewski, Robert D. Tilton, Gregory V. Lowry

**Affiliations:** ^†^Department of Civil and Environmental Engineering, ^‡^Center for Environmental Implications of Nano Technology (CEINT), ^§^Department of Chemistry, ^∥^Department of Chemical Engineering, and ^⊥^Department of Biomedical Engineering, Carnegie Mellon University, Pittsburgh, Pennsylvania 15213, United States; # Department of Botany and Plant Sciences, University of California, Riverside, California 92521, United States; ○ Department of Civil and Environmental Engineering, Massachusetts Institute of Technology, Cambridge, Massachusetts 02139, United States

**Keywords:** star polymers, bottlebrush polymers, foliar application, phloem loading, agrochemical delivery

## Abstract

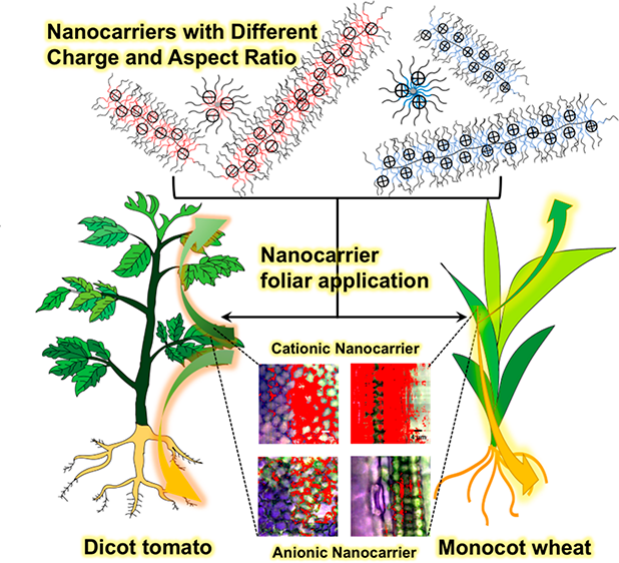

An incomplete understanding of how agrochemical nanocarrier properties affect their uptake and translocation in plants limits their application for promoting sustainable agriculture. Herein, we investigated how the nanocarrier aspect ratio and charge affect uptake and translocation in monocot wheat (*Triticum aestivum*) and dicot tomato (*Solanum lycopersicum*) after foliar application. Leaf uptake and distribution to plant organs were quantified for polymer nanocarriers with the same diameter (∼10 nm) but different aspect ratios (low (L), medium (M), and high (H), 10–300 nm long) and charges (−50 to +15 mV). In tomato, anionic nanocarrier translocation (20.7 ± 6.7 wt %) was higher than for cationic nanocarriers (13.3 ± 4.1 wt %). In wheat, only anionic nanocarriers were transported (8.7 ± 3.8 wt %). Both low and high aspect ratio polymers translocated in tomato, but the longest nanocarrier did not translocate in wheat, suggesting a phloem transport size cutoff. Differences in translocation correlated with leaf uptake and interactions with mesophyll cells. The positive charge decreases nanocarrier penetration through the leaf epidermis and promotes uptake into mesophyll cells, decreasing apoplastic transport and phloem loading. These results suggest design parameters to provide agrochemical nanocarriers with rapid and complete leaf uptake and an ability to target agrochemicals to specific plant organs, with the potential to lower agrochemical use and the associated environmental impacts.

## Abstract 2

The charge and aspect ratio of polymer nanocarriers affect leaf uptake and translocation in crop plants, providing design rules for nanocarriers for efficient and targeted agrochemical/biomolecule delivery.

## Introduction

Improving agrochemical utilization efficiency and climate resilience of crop plants is critical for global food security and reducing the environmental impacts of agriculture. Both increasing agrochemical use efficiency and developing climate resilient plants through genetic engineering require methods for efficient and targeted agent/biomolecule delivery to plants.^1,2^ Agrochemical uptake and use efficiency for state of the art methods are low for applied active ingredients in pesticides (<0.1%) and micronutrients (<5%).^3−6^ Global agrochemical annual use is staggering, including 1.2 × 10^8^ t (metric tonnes) for N-based fertilizers, 5 × 10^7^ t for phosphate-based fertilizers, and over 2.6 × 10^6^ t for pesticides.^7^ The total embodied energy for agrochemical use is ∼3.1 GJ/ha.^8^ Global agricultural land area is approximately 5 billion hectares, translating to ∼1.55 × 10^11^ GJ of energy used for agrochemical production, or ∼30% of global energy consumption.^9^ Efficient and targeted agrochemical delivery could lead to significant energy savings from lower agrochemical use and reduced environmental impacts from excess agrochemical discharge. Low agent delivery efficiency also results in inefficient gene delivery to plants that limit plant genetic engineering.^10^ New agent delivery approaches are needed to improve efficiency, increase crop climate resilience, and promote sustainability of agriculture.

Foliar application of agrochemical loaded nanocarriers can promote efficient agent delivery into plants.^4^ Nanoparticles and biomolecular and polymeric nanocarriers with various properties have been foliar applied to plants and demonstrated significant uptake and translocation.^11−14^ Gold nanoparticles with sizes ranging from 3 to 50 nm had nearly complete (100%) uptake into wheat leaves and up to ∼60% of the applied particles translocated from the exposed leaf to various plant organs.^12^ Star polymer based nanocarriers with different sizes, charges, and hydrophobicity also had high (∼100%) uptake and up to ∼30% total translocation away from the leaf after foliar exposure.^11^ Approximately 100 nm polypeptide nanoparticles and polymer functionalized carbon nanotubes have delivered nucleic acids into specific cell organelles such as the cell nucleus and chloroplasts to transfect plants after foliar application.^10,14−18^ Mesoporous silica nanoparticles have delivered micronutrients and active ingredients into tomato plants with significant (∼20%) phloem loading and translocation.^19,20^ These studies all indicate that foliar applied nanomaterials can pass through physical barriers in plants and potentially achieve higher agent delivery efficiency compared to conventional agrochemical application techniques.^21^

While nanomaterial uptake and translocation in plants have been recently studied, the factors that direct the uptake and systemic translocation of nanomaterials in plants remain unclear.^22^ Both nanomaterial properties and plant leaf biosurface and anatomy can affect nanomaterial uptake and transport.^23,24^ Nanoparticle charge can play an important role in their uptake through plant cell organelle lipid membranes, but the effects can depend on the delivery method used.^21^ For example, using foliar application of surfactant-based formulations, positively charged nanoparticles had 30% greater colocalization with chloroplasts of crop plants than their anionic counterparts.^23^ However, when delivering nanoparticles using pressure driven leaf lamina infiltration, positively charged nanoparticles had 20% lower colocalization with chloroplasts of *Arabidopsis thaliana* plants compared to negatively charged ones.^24^ Nanoparticle size also plays a role in their distribution in different plant organs after foliar application. Smaller ∼5 nm star polymers (polymer nanocarriers) translocated preferentially to younger leaves, while larger ∼35 nm star polymers mainly accumulated in roots of tomato plants.^11^ The smaller size of star polymers may favor their phloem unloading into nonvascular tissues of leaves, thereby favoring their distribution to younger and older leaves, while the large size polymers may inhibit phloem unloading and phloem to xylem exchange, leading to greater accumulation of the larger star polymer in roots.^11^ Plant leaf anatomy (monocot vs dicot) also affects nanoparticle uptake and translocation in plants.^25,26^ Maize, a monocot plant, mainly takes up foliar applied nanoparticles through stomata, whereas cotton, a dicot plant, takes up nanoparticles through both stomata and cuticular pathways.^25^ Different plant species also have a wide range of leaf surface pH (5–10),^27^ leading to different phyllosphere microbiomes that could potentially interact differently with applied nanomaterials and change their uptake pathways. Although these previous studies have investigated factors that direct nanomaterial interactions with specific plant organelles,^6,23,28−30^ the effect of the aspect ratio and charge on leaf uptake after foliar spray (the only practical method for field application) and subsequent systemic translocation and distribution in plants with different leaf anatomy remains to be elucidated. An incomplete understanding of how a nanomaterial’s properties affect uptake and systemic translocation in plants remains an obstacle for designing materials for efficient foliar application.^22^

To elucidate how the charge and aspect ratio direct nanocarrier leaf uptake and translocation in important crop plants, we designed and synthesized gadolinium (Gd)- or dye-loaded polymer based nanocarriers with identical composition but different aspect ratios. This includes a 21-armed nominally spherical star polymer with a low aspect ratio of 1.1 (denoted as ‘L’), a short bottlebrush with a medium aspect ratio of 8.2 (denoted as ‘M’), and a long bottlebrush polymer with a high aspect ratio of 28.5 (denoted as ‘H’). To determine the influence of the charge, the polymer core was either negatively charged polyacrylic acid (PAA) or positively charged poly(2-(dimethyl amino)ethyl methacrylate) (PDMAEMA), but the polymer structures were the same to yield anionic (denoted as L–, M–, H−) or cationic (denoted as L+, M+, H+) nanocarriers with three different aspect ratios. Their phloem loading and distribution in different plant organs in monocot (wheat) and dicot (tomato) plants were assessed by inductively coupled plasma mass spectrometry (ICP-MS). The nanocarrier leaf uptake pathway and interactions with plant mesophyll cells were visualized by Hyperspectral-Enhanced Dark Field Microscopy (DF-HSI). This study revealed the important nanocarrier design parameters that direct their uptake, translocation in different crop species, and how the nanocarrier-plant cell interactions directed nanocarrier translocation in plants.

## Experimental Section

### Materials

*N*-Isopropylacrylamide (NIPAm, 97%), 2-(dimethyl amino)ethyl methacrylate (DMAEMA, 98%), β-cyclodextrin (β-CD), 2-bromoisobutyryl bromide (BiBB, 98%), 1-methyl-2-pyrrolidone (NMP), 1,1,4,7,10,10-hexamethyltriethylenetetramine (HMTETA, 97%), dichloromethane (DCM), copper powder (Cu^0^, 99.7%, 45 cm^2^ g^–1^), Rose Bengal (RB, 95%), Crystal violet (CV, ≥90.0%), diethylenetriaminepentaacetic acid gadolinium(III) dihydrogen salt (Gd-DTPA, 97%), ethyl 2-bromoisobutyrate (EBiB, 98%), copper(I) bromide (CuBr, ≥99.95%), copper(II) bromide (CuBr_2_, ≥99.995%), potassium fluoride (KF, 99%), basic alumina, and chloroform-d (CDCl_3_) were obtained from Sigma-Aldrich. (2-Trimethylsiloxy)ethyl methacrylate (HEMA-TMS) was purchased from Scientific Polymer Products. Tris(2-dimethylaminoethyl) amine (Me_6_TREN, ≥99%), gadolinium(III) chloride hexahydrate (GdCl_3_·6H_2_O, 99%), anisole (99%), and *N*,*N*-dimethylformamide (DMF, 99%) were purchased from Alfa Aesar. HNO_3_ (70%, trace metal grade), Silwet L-77, and H_2_O_2_ (30%, ACS grade) were purchased from Fisher Scientific. Dialysis bags with desired molecular weight cutoffs were purchased from Spectrum lab (Spectra/Por 7). The DMAEMA monomer was purified by passing it through basic alumina to remove the inhibitor. Other chemicals were used as-received without further purification.

### Synthesis of 21-Armed PDMAEMA_50_-*b*-PNIPAm_50_ Star Polymers (L+)

*β-*Cyclodextrin was functionalized with 2-bromoisobutyryl bromide (BiBB) to synthesize the β-CD-21Br initiator following our previous study.^11^ The PDMAEMA block of the star polymer was prepared by normal atom transfer radical polymerization (ATRP) (Figure S1). Briefly, 0.05 g (1 equiv) of β-CD-21Br initiator, 10.4 mL (5250 equiv) of DMAEMA, 6.6 mg (4.2 equiv) of CuCl_2_, 0.067 mL (21 equiv) of HMTETA, and 20.7 mL of anisole were mixed in a sealed Schlenk flask equipped with a stir bar. The Schlenk flask was degassed by purging with N_2_ for 40 min, and the reaction was frozen by plunging it into liquid nitrogen. The flask was opened briefly to add 24.4 mg (21 equiv) of CuCl powder to the frozen reaction. The flask was sealed again and purged with N_2_ for 30 min before being allowed to warm to room temperature. The reaction was stopped at 20% conversion to yield a 21 armed PDMAEMA star polymer with 50 DMAEMA repeating units in each arm. The products were purified by dialysis (MWCO = 8000 Da) against methanol for 3 cycles. The molecular weight distribution of the PDMAEMA star polymer was measured by gel permeation chromatography with multiangle laser light scattering (GPC-MALLS) (Table S1).

The PNIPAm chain extension procedure was adapted from a previously reported procedure (Figure S1).^4^ Briefly, 0.4 g (1 equiv) of the as-synthesized PDMAEMA star polymer, 0.56 g (2100 equiv) of NIPAm, 0.55 mg (1.05 equiv) of CuBr_2_, 0.0013 mL (2.1 equiv) of Me_6_Tren, and 5.6 mL of DMF were mixed in a sealed Schlenk flask equipped with a stir bar. The Schlenk flask was degassed by purging with N_2_ for 40 min, and the reaction was frozen by plunging it into liquid nitrogen. The flask was opened briefly to add 0.084 g (0.68 cm^–1^) of Cu^0^ powder. The flask was sealed again and purged with N_2_ for 30 min before being allowed to warm to room temperature. The reaction was constantly monitored by ^1^H NMR and stopped at 50% conversion to yield PDMAEMA_50_-*b*-PNIPAm_50_ star polymers (L+). The reaction mixture was first filtered by a column filled with cotton to remove the Cu powder and then dialyzed against methanol for 3 cycles (MWCO = 8000 Da). The chemical composition of the product was verified by ^1^H NMR in CDCl_3_ (Figure S2a). Detailed synthesis procedures of other star and bottlebrush polymers used in this study and their characterization procedures are similar and are provided in the Supporting Information.

### Plant Growth

The tomato (*Solanum lycopersicum*, Roma VF) and wheat (*T. aestivum*, Cumberland) plants used in this study were cultured in quarter strength Hoagland’s solution aerated using air pumps. While hydroponically grown plants have easy access to necessary nutrients that may affect their phloem transport, the trends in nanomaterial uptake and transport in hydroponic plants are generally similar to soil cultured plants,^11,12^ and we expect the results measured this way are representative of soil grown plants, especially for foliar applied nanoparticles. The tomato and wheat seeds were rinsed twice by Milli-Q water before being sterilized with 10% bleach for 1 min and rinsed again by Milli-Q water 5 times. The sterilized seeds were germinated on water-moistened filter paper in a Petri dish for 10 days in the dark. The seedlings were then transplanted to 100 mL plastic cups covered with a plastic lid with five holes. Four seedlings were transplanted to each cup with roots suspended in Hoagland solution. Plants were grown at 22 °C with a 16-h light and 8-h dark cycle for 30 days before nanocarrier foliar exposure.

### Polymer Nanocarrier Foliar Application, Uptake, and Transport in Wheat and Tomato Plants

Gd^3+^ or gadolinium-diethylenetriaminepentaacetic acid (Gd-DTPA) was used to label the anionic and cationic polymer nanocarriers. Stability of the Gd-nanocarrier complex was assessed by dialysis in simulated apoplastic fluid.^11,13^ The Gd loading in the different nanocarriers is shown in Table S4. Between 0.7 and 2.8% (most <1%) of the loaded Gd leached out of nanocarriers in simulated apoplastic fluid after 24 h (Table S5), suggesting that the Gd labeling in the nanocarriers is stable in plants and the Gd detected in different plant tissues is associated with polymers.^11^ The detailed Gd loading procedure is provided in the SI.

The Gd loaded polymer nanocarriers with 1 g L^–1^ polymer concentration were applied to the second true leaf of tomato plants or the third leaf of wheat plants with 0.1 vol % Silwet L-77 surfactant, an agricultural wetting agent commonly used in agrochemical sprays.^25^ The polymer solutions were applied as 4 drops of 5 μL each (20 μg of polymer total) on the adaxial (top) side of the leaf. Each treatment had 5 replicate plants. Plants were harvested 3 days after treatment and cut into 5 parts: leaf where the Gd-loaded polymer solutions were applied (denoted as “exposed zone”), leaves at growth stages lower than the exposed leaves (denoted as “younger leaf”), leaves at growth stages higher than exposed leaves (denoted as “older leaf”), stem of the plant (denoted as “stem”), and roots (denoted as “root”). A control experiment was conducted by applying a GdCl_3_ salt at 200 mg L^–1^ Gd concentration to wheat plants (Figure S6h). The free Gd control experiments in tomato were conducted in a previous study.^4^ Total transport of nanocarriers was defined as the total fraction of the applied nanocarrier that moved out of the exposed leaf and into other plant organs and is calculated by the total mass of Gd detected in younger and older leaves, stem, and root over the total mass of Gd applied.

To quantify polymer nanocarrier distribution in different plant organs, the Gd content in different plant tissues was measured by ICP-MS. All plant samples were dried at 105 °C for 48 h to dehydrate them. The dried plant samples were digested overnight with a 1-mL mixture of 2:1 v/v 70% HNO_3_ and 30% H_2_O_2_ at room temperature, followed by heating at 100 °C for 45 min. Post digestion, samples were diluted to 5% HNO_3_ by Milli-Q water and filtered by a 0.45 μm PTFE syringe filter before being analyzed by ICP-MS.^12^ The Gd recovery, calculated from the mass of Gd detected by ICP-MS over the Gd mass applied to plants, is 69.7–107.1% (Table S6). The Gd calibration curve acquired by ICP-MS is reported in Table S7.

### Imaging Polymer-Leaf Interactions

The nanocarriers were labeled with organic dyes with strong light absorbance to track their distribution in plant leaves using hyperspectral imaging. The polymer nanocarriers were labeled by organic dyes through electrostatic attraction. This labeling could slightly neutralize the charge of nanocarriers but would not substantially change the size and charge of nanocarriers as indicated in our previous publication^11,31^ and thus would not significantly affect their interaction with cells. The organic dye Rose Bengal (RB) and Crystal violet (CV) labeling procedure is documented in the SI. The RB or CV labeled polymer nanocarriers were applied to wheat or tomato leaves with or without 0.1 vol % Silwet L-77. Four drops of a 5-μL dye labeled polymer solution at 1 g L^–1^ polymer concentration were applied to each leaf. The exposed leaves were incubated for 24 h before being imaged using a hyperspectral imaging system (CytoViva, Inc.) as previously described.^13^ The focal planes for hyperspectral images included the epidermis cell layer, mesophyll cells, and leaf cross sections. Detailed information on spectral library development is described in the SI. The spectral library (Figure S4) was used to identify dye labeled polymer nanocarriers in leaves to map their distribution in exposed leaves using spectral angle mapping (SAM, ENVI 5.2).

## Results and Discussion

### Characteristics of Polymer Nanocarriers with Different Aspect Ratios

Polymer nanocarriers with different charges and aspect ratios, including low aspect ratio star polymers (L), medium aspect ratio short bottlebrush (M), and high aspect ratio long bottlebrush (H) polymers, were synthesized by a grafting from approach (Figure S1).^32,33^ The chemical composition of cationic star and bottlebrushes was confirmed by ^1^H NMR (Figure S2) and gel permeation chromatography with a multiangle laser light scattering detector (GPC-MALLS), as shown in Figure S3 and Table S1. The theoretical molecular weight (*M*_n_) of polymer bottlebrushes and star polymers calculated from ^1^H NMR and GPC-MALLS ranges from 1.76 × 10^5^ to 2.20 × 10^7^ g mol^–1^, as shown in Table S2. The anionic polymer nanocarriers with PAA_50_-*b*-PNIPAm_50_ diblock copolymer arms, including anionic star polymers (L−), short anionic bottlebrush (M−), and long anionic bottlebrush (H−) polymers, were synthesized and characterized previously.^4^

**Figure 1:**
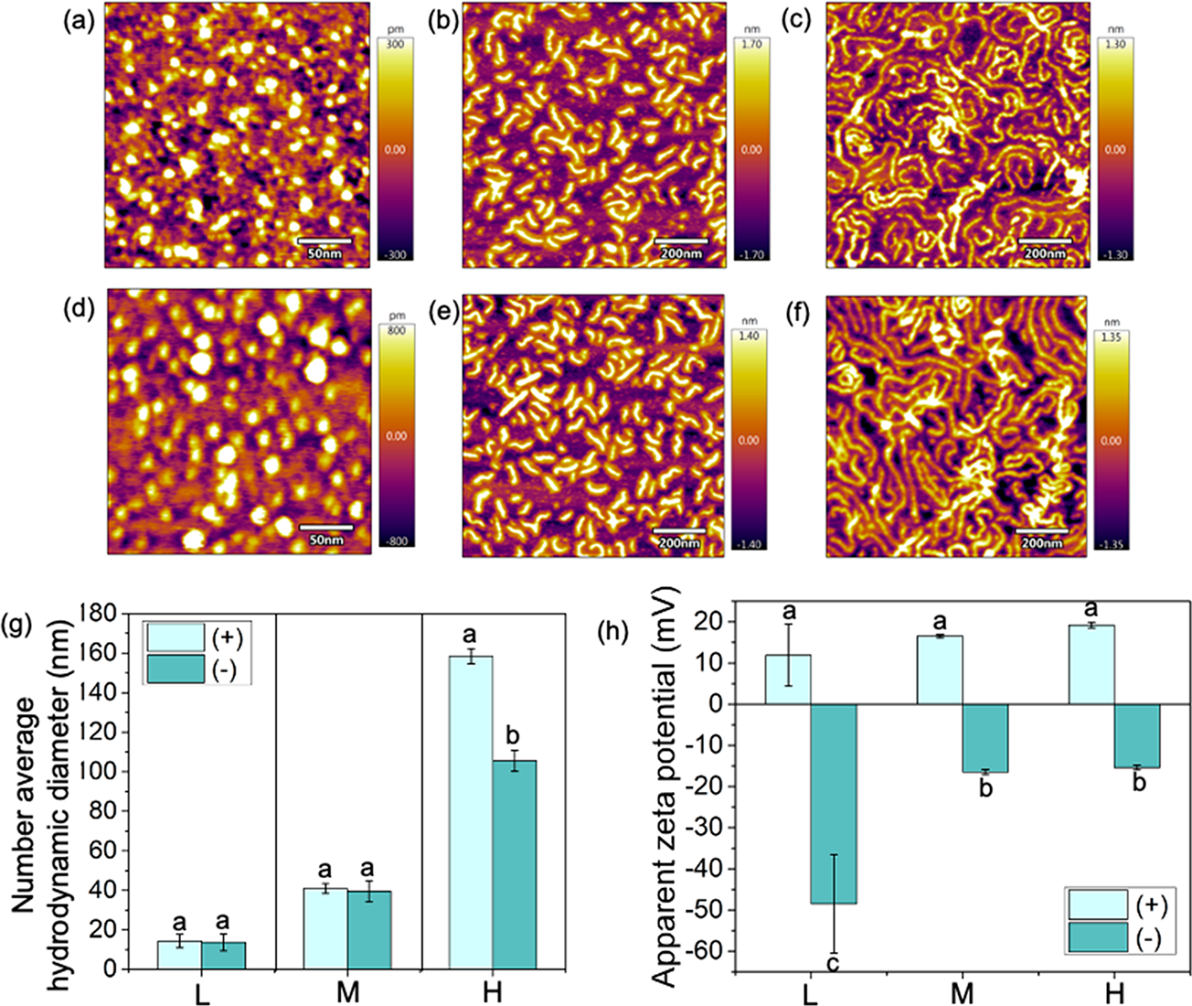
Atomic force microscope height images of (a) 21-armed PDMAEMA_50_-*b*-PNIPAm_50_ star polymer (L+), (b) P[BiBEM-*g*-(PDMAEMA_50_-*b*-PNIPAm_50_)]_320_ short polymer bottlebrush (M+), and (c) P[BiBEM-*g*-(PDMAEMA-*b*-PNIPAm)] long polymer bottlebrush (H+). (d) PAA_50_-*b*-PNIPAm_50_ star polymer (L−), (e) [BiBEM-*g*-(PAA_50_-*b*-PNIPAm_50_)]_320_ short polymer bottlebrush (M−), and (f) P[BiBEM-*g*-(PAA-*b*-PNIPAm)] long polymer bottlebrush (H−). (g) Number-average hydrodynamic diameter based on dynamic light scattering measurements and (h) apparent zeta potentials (ζ) of L+, M+, H+, L–, M–, and H– determined in water at a 100 mg L^–1^ polymer concentration at pH 6.5 in 10 mM NaCl by dynamic light scattering (Malvern Zetasizer Nano ZS) and the Smoluchowski approximation. Error bars represent standard deviation (*n* = 3).

The morphologies of the nanocarriers were assessed by atomic force microscopy. L+ are spherical with an ∼10 nm diameter (Figure 1a). Bottlebrush polymers are high aspect ratio rod-like structures, with a length of ∼80 nm for M+ (Figure 1b) and around 300 nm for H+ (Figure 1c). The morphologies of the anionic nanocarriers were similar to that of cationic carriers (Figure 1d,e,f). The polymer nanocarriers with different charges but the same number of arms have comparable hydrodynamic diameters (Figure 1g). The number-average diameter of L+ and L– is 14.3 ± 3.5 nm and 13.7 ± 4 nm, respectively (Figure 1g, Table S2). The short polymer bottlebrushes have an average hydrodynamic diameter of 40.9 ± 2.6 nm for M+ and 39.5 ± 5.4 nm for M– (Figure 1g, Table S2). The long 1600-armed bottlebrushes have hydrodynamic diameters over 100 nm, with 158.4 ± 3.8 nm for H+ and 105.5 ± 5.3 nm for H– (Figure 1g, Table S2). The apparent zeta potential of cationic nanocarriers with PDMAEMA-*b*-PNIPAm arms varied from 11.9 ± 7.5 mV to 19.2 ± 0.7 mV, while the apparent zeta potential for anionic nanocarriers with PAA-*b*-PNIPAm arms varied from −48.5 ± 12 mV to −15.4 ± 0.6 mV (Figure 1h, Table S2). The aspect ratios of L+, M+, and H+ are 1.1 ± 0.3, 8.2 ± 2.5, and 28.5 ± 9.8, respectively, as analyzed from their AFM height images (Table S3). Similar results were obtained for the anionic materials. Thus, comparisons of their behaviors allow for a detailed assessment of how the charge (+ vs −) and aspect ratio are directing their uptake and translocation in plants.

### Effect of the Charge on Polymer Nanocarrier Translocation and Distribution in Tomato

**Figure 2:**
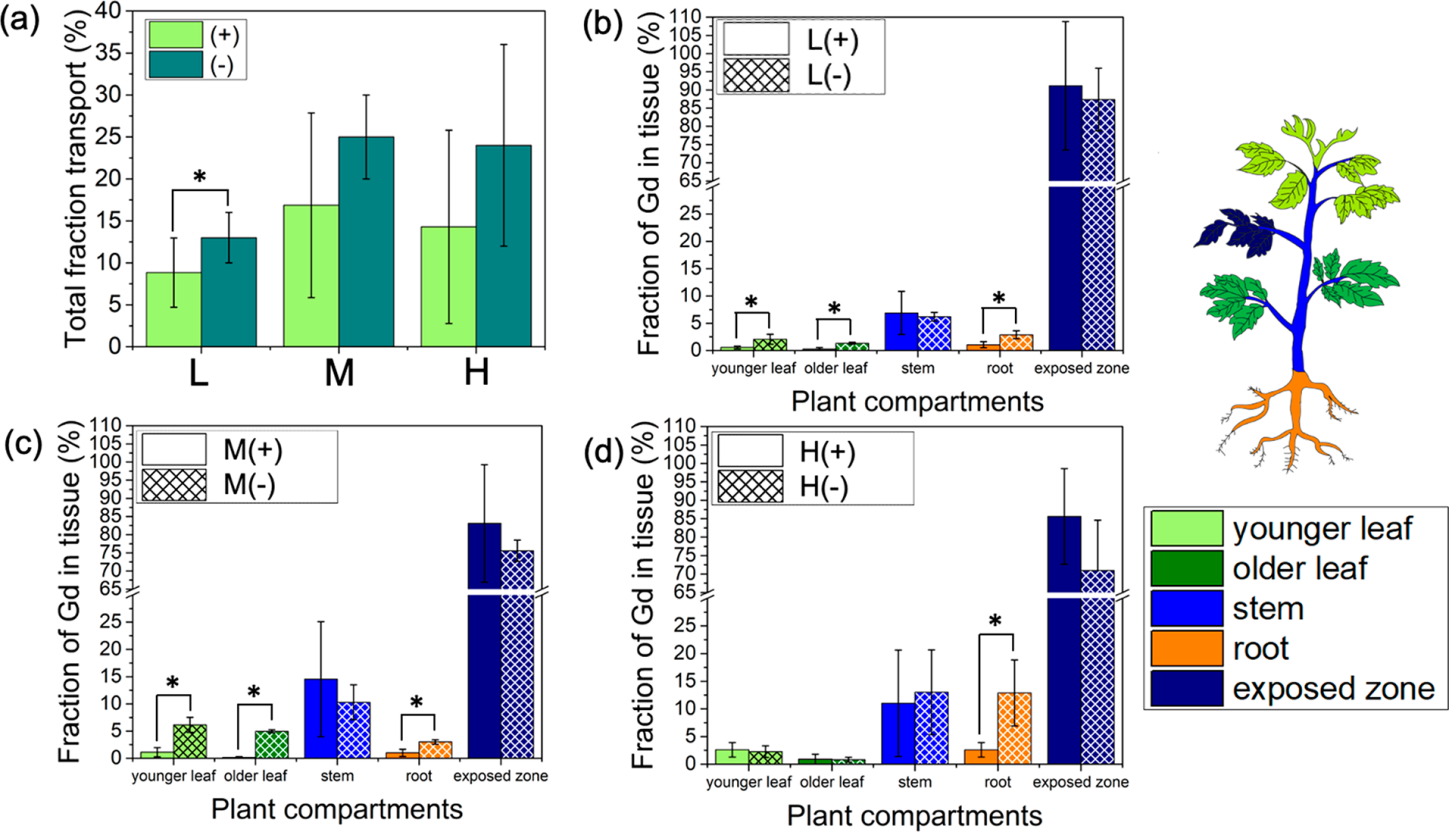
Effect of polymer nanocarrier charge on their uptake and total translocation in tomato plants. (a) Total fraction of Gd loaded nanocarriers transported out of exposed leaf to other plant organs in tomato plants. (b) Distribution of PDMAEMA_50_-*b*-PNIPAm_50_ (L+) and PAA_50_-*b*-PNIPAm_50_ (L−) spherical star polymers with different charges, (c) P[BiBEM-*g*-(PDMAEMA_50_-*b*-PNIPAm_50_)]_320_ (M+) and P[BiBEM-*g*-(PAA_50_-*b*-PNIPAm_50_)]_320_ (M−) short bottlebrushes with different charges, (d) [BiBEM-*g*-(PDMAEMA_50_-*b*-PNIPAm_50_)]_1600_ (H+) and P[BiBEM-*g*-(PAA_50_-*b*-PNIPAm_50_)]_1600_ (H−) long bottlebrushes with different charges. Error bars represent standard deviation (*n* = 5–6). ANOVA test followed by Fisher’s LSD test was used for multiple comparisons, *P ≤ 0.05. Statistical analysis was performed between cationic and anionic nanocarriers in the same plant organ.

Nanocarrier charge played an important role in their phloem loading and distribution in the model dicot, tomato. The cationic nanocarriers, including L+, M+, and H+, had ∼13 ± 4% total transport (Figure 2a, Figure S6). The anionic nanocarriers had higher transport in tomato plants with ∼20.7 ± 6.7% total transport (Figure 2a). Both the anionic and cationic carriers transported more than free Gd applied to the tomato leaves (<1%),^4^ indicating that the nanocarriers are facilitating Gd uptake and translocation to different plant organs. While the total transport of anionic nanocarriers is higher than for cationic nanocarriers in tomato plants, they are not statistically significantly different at 95% confidence (*p* < 0.05) due to a relatively high variation between biological replicates (Figure 2a).

In addition to total transport, nanocarrier charge also affected their distribution to different tomato plant organs. The negatively charged low and medium aspect ratio materials (L– and M−) had higher accumulation in younger leaves, older leaves, and roots compared with their positively charged counterparts, L+ and M+ (Figure 2b,c). Over 5% of applied M– transported to younger and older mature leaves, suggesting that M– can be used to deliver agents into different leaves (Figure 2c). Mature older leaves stop importing phloem materials from other plant organs and only export photosynthesis products through phloem.^34^ Therefore, the mature older leaves can only take up nanocarriers through xylem.^11^ These results suggest that negatively charged L– and M– are exchanged from phloem to xylem sap and moved to older leaves through xylem flow, but the positively charged ones are not. Around 13% of the high aspect ratio anionic bottlebrush (H−) was found in plant roots, while H+ only had significant transport to stems (Figure 2d). These results suggest the positively charged nanocarriers are best suited for delivery to the plant stem, whereas the negatively charged carriers are the most suitable nanocarrier for delivery to other plant organs and roots in dicot plants.

### Effect of the Charge on Polymer Nanocarrier Translocation and Distribution in Wheat

**Figure 3:**
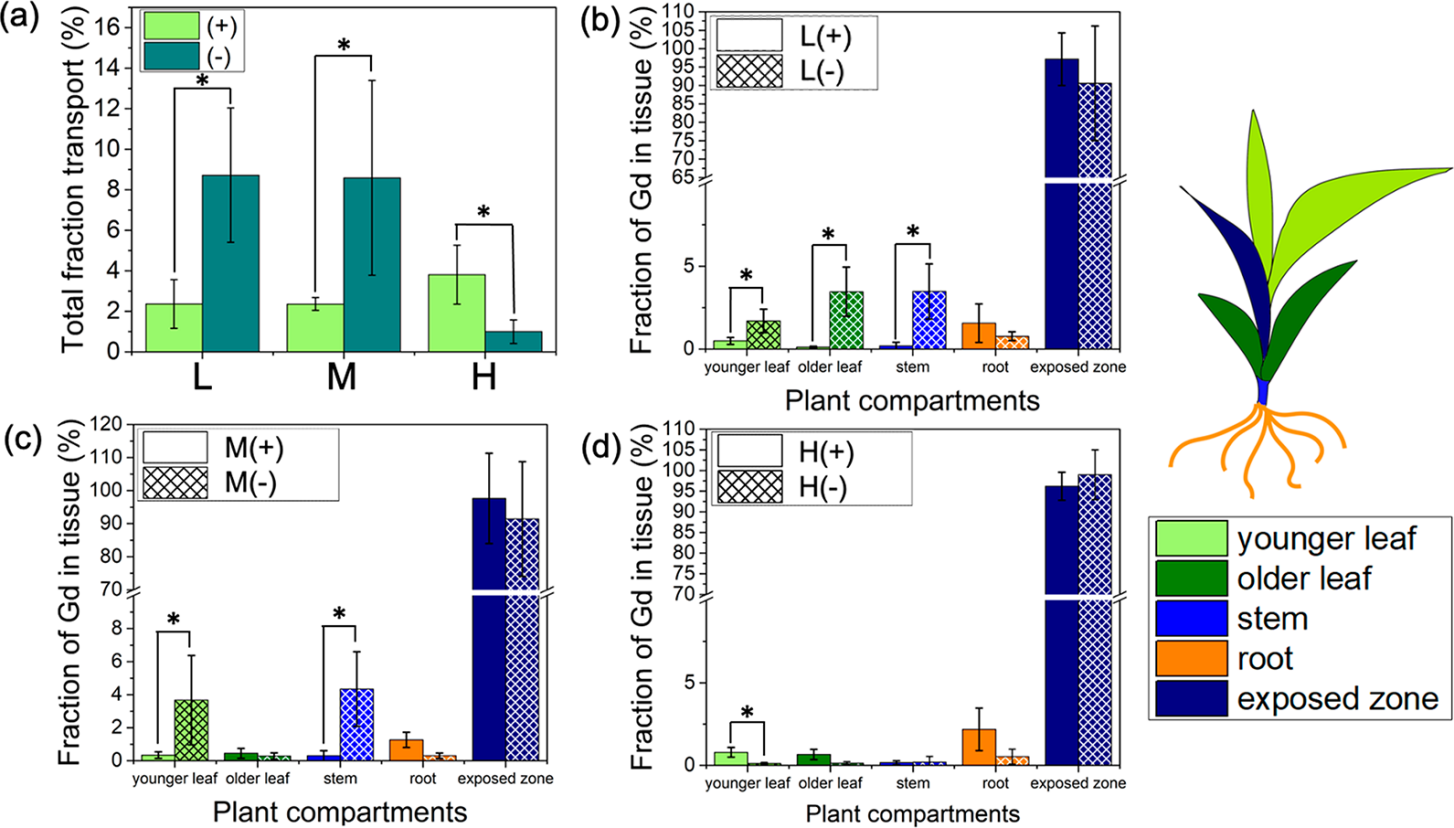
Effect of polymer nanocarrier charge on their uptake and total translocation in wheat plants. (a) Total fraction of Gd loaded nanocarriers transported out of exposed leaf to other plant organs in wheat plants. (b) Distribution of PDMAEMA_50_-*b*-PNIPAm_50_ (L+) and PAA_50_-*b*-PNIPAm_50_ (L−) spherical star polymers with different charges, (c) P[BiBEM-*g*-(PDMAEMA_50_-*b*-PNIPAm_50_)]_320_ (M+) and P[BiBEM-*g*-(PAA_50_-*b*-PNIPAm_50_)]_320_ (M−) short bottlebrushes with different charges, (d) [BiBEM-*g*-(PDMAEMA_50_-*b*-PNIPAm_50_)]_1600_ (H+) and P[BiBEM-*g*-(PAA_50_-*b*-PNIPAm_50_)]_1600_ (H−) long bottlebrushes with different charges. Error bars represent standard deviation (*n* = 5–6). ANOVA test followed by Fisher’s LSD test was used for multiple comparisons, *P ≤ 0.05. Statistical analysis was performed between cationic and anionic nanocarriers in the same plant organ.

Nanocarrier charge also affected their translocation and distribution in the model monocot, wheat (Figure 3). Only ∼2.9 ± 0.8% of the cationic L+, M+, and H+ nanocarriers translocated away from the exposed leaf in wheat plants (Figure 3a, Figure S6d), only slightly higher than the free Gd transport in wheat (0.96 ± 0.33%, Figure S6h), suggesting the cationic nanocarriers do not readily access phloem and transport in monocot wheat plants. The anionic nanocarriers, including L– and M–, had greater (∼8.7%) total transport than their cationic counterparts. However, the highest aspect ratio negatively charged nanocarrier (H^–^) did not translocate from the exposed leaf (Figure 3a).

The charge also affected nanocarrier distribution in wheat plants. The anionic L– had greater transport to younger leaves, older leaves, and stems compared with L+ (Figure 3b); M– also accumulated more in younger leaves and stems compared with M+ but not roots (Figure 3c). Both H+ and H– had limited translocation in wheat to all plant organs (Figure 3d). The general trends in wheat are similar to tomato, with cationic nanocarriers showing lower phloem loading and transport compared with their anionic counterparts. This is likely because of stronger interaction between cationic nanocarriers and the negatively charged plant cell membranes that caused them to be kinetically trapped in cell organelles and lowered their mobility in plants.^25^ The charge of nanocarriers may also affect their interaction with proteins, resulting in a different protein corona composition and thickness that may drive nanocarrier translocation and distribution in plants.^35^

### Effect of the Aspect Ratio on Polymer Nanocarrier Translocation and Distribution

**Figure 4:**
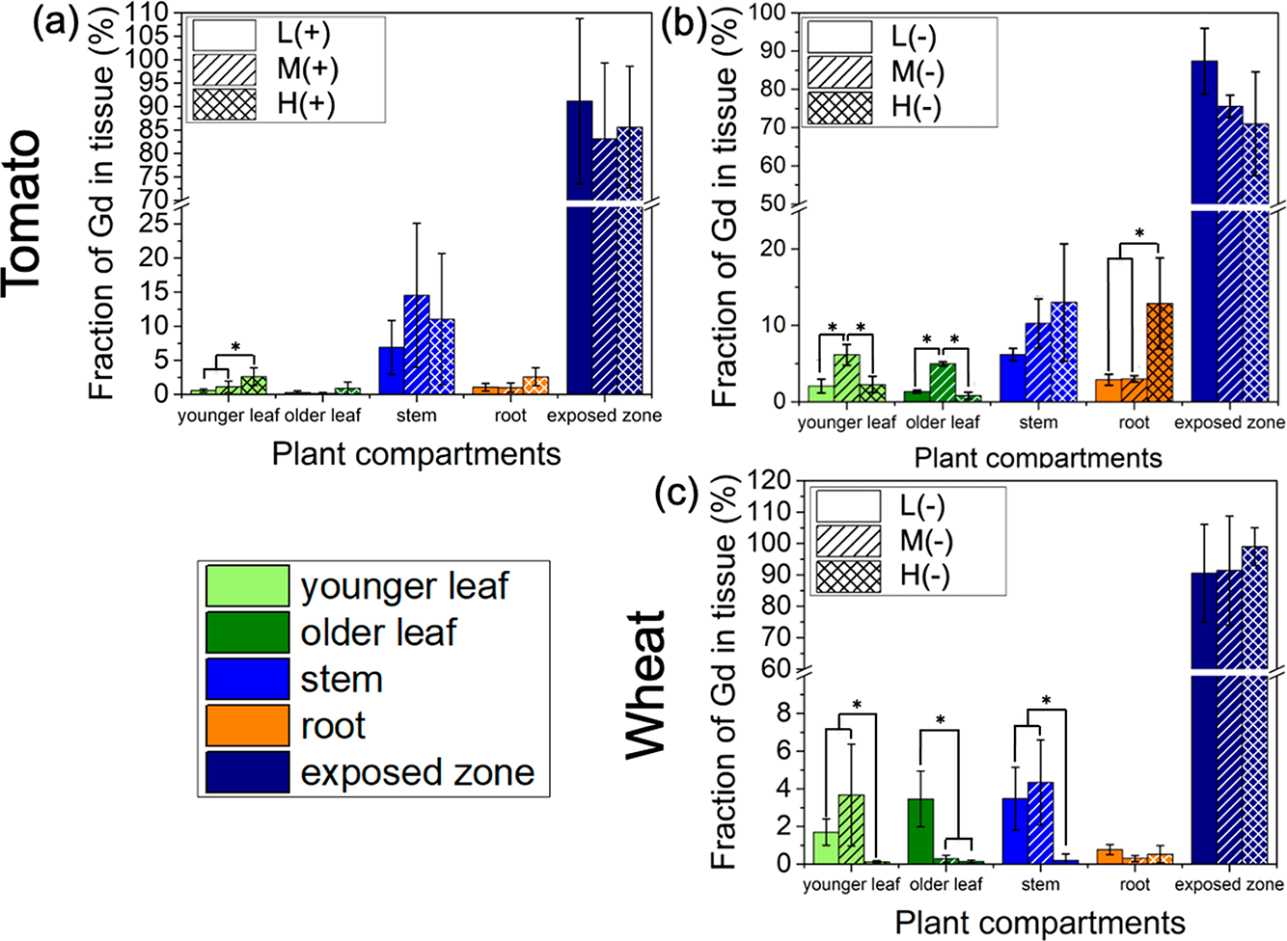
Effect of the polymer nanocarrier aspect ratio on their uptake and translocation in tomato and wheat plants. (a) PDMAEMA_50_-*b*-PNIPAm_50_ star (L+), P[BiBEM-*g*-(PDMAEMA_50_-*b*-PNIPAm_50_)]_320_ (M+), and P[BiBEM-*g*-(PDMAEMA_50_-*b*-PNIPAm_50_)]_1600_ (H+) positively charged nanocarriers with different aspect ratios in tomato plants. (b) PAA_50_-*b*-PNIPAm_50_ (L−), P[BiBEM-*g*-(PAA_50_-*b*-PNIPAm_50_)]_320_ (M−), and P[BiBEM-*g*-(PAA_50_-*b*-PNIPAm_50_)]_1600_ (H−) negatively charged nanocarriers with different aspect ratios in tomato plants. (c) PAA_50_-*b*-PNIPAm_50_ (L−), P[BiBEM-*g*-(PAA_50_-*b*-PNIPAm_50_)]_320_ (M−), and P[BiBEM-*g*-(PAA_50_-*b*-PNIPAm_50_)]_1600_ (H−) negatively charged nanocarriers in wheat plants. Error bars represent standard deviation (*n* = 5–6). ANOVA test followed by Fisher’s LSD test was used for multiple comparisons, *P ≤ 0.05. Statistical analysis was performed between different aspect ratio nanocarriers in the same plant organ.

Contrary to the expectation that the larger hydrodynamic diameter of the higher aspect ratio particles would inhibit their translocation in plants, the nanocarrier aspect ratio had only a limited impact on the amount of the applied nanocarrier that translocated to other plant organs in tomato plants. In tomato, the total mass of the translocated polymer was not statistically significantly different between the aspect ratios used (1.1, 8.2, and 28.5). However, in wheat, the highest aspect ratio material (H^–^) did not translocate, suggesting that there could be a size cutoff in monocots that does not exist in dicots (Figure 4). This provides an upper limit for nanocarrier use size in monocots. One benefit of using high aspect ratio materials is that they can hold a larger amount of agrochemical per applied nanocarrier polymer and therefore deliver more of an active ingredient into cells or organelles on a per carrier basis than lower aspect ratio materials.^36^ This may increase efficacy for applications where particle uptake is the rate limiting step, e.g. delivery of DNA into chloroplasts, mitochondria, or the cell nucleus.

While the nanocarrier aspect ratio did not affect total translocation, it did affect the distribution to other plant organs in both wheat and tomato plants for anionic nanocarriers. For anionic nanocarriers in tomato, M– showed higher transport into younger and older leaves, whereas H– exhibited higher (∼13%) accumulation in roots (Figure 4b). These results suggest the larger hydrodynamic diameter of H– may inhibit phloem to xylem exchange and their translocation to younger leaves, causing more nanocarrier to be trapped in roots.^11^ This is consistent with a previous study where spherical polymer nanoparticles with a larger hydrodynamic diameter tended to accumulate in plant roots.^11,12^ The nanocarrier aspect ratio also affects their distribution in monocot wheat plants (Figure 4c). L– mainly translocated into younger leaves, older leaves, and stems, whereas M– only translocated to younger leaves and stems but not to older leaves (Figure 4c). This suggests that the higher aspect ratio of M– did not favor their transport to older leaves through phloem and xylem exchange. In contrast to anionic nanocarriers, the aspect ratio did not significantly affect cationic nanocarrier distribution in either tomato and wheat plants, as the transport efficiency is generally low (<10%) for cationic nanocarriers (Figure 2a, Figure 3a).

### Polymer Nanocarrier Interaction with Plant Leaves

**Figure 5:**
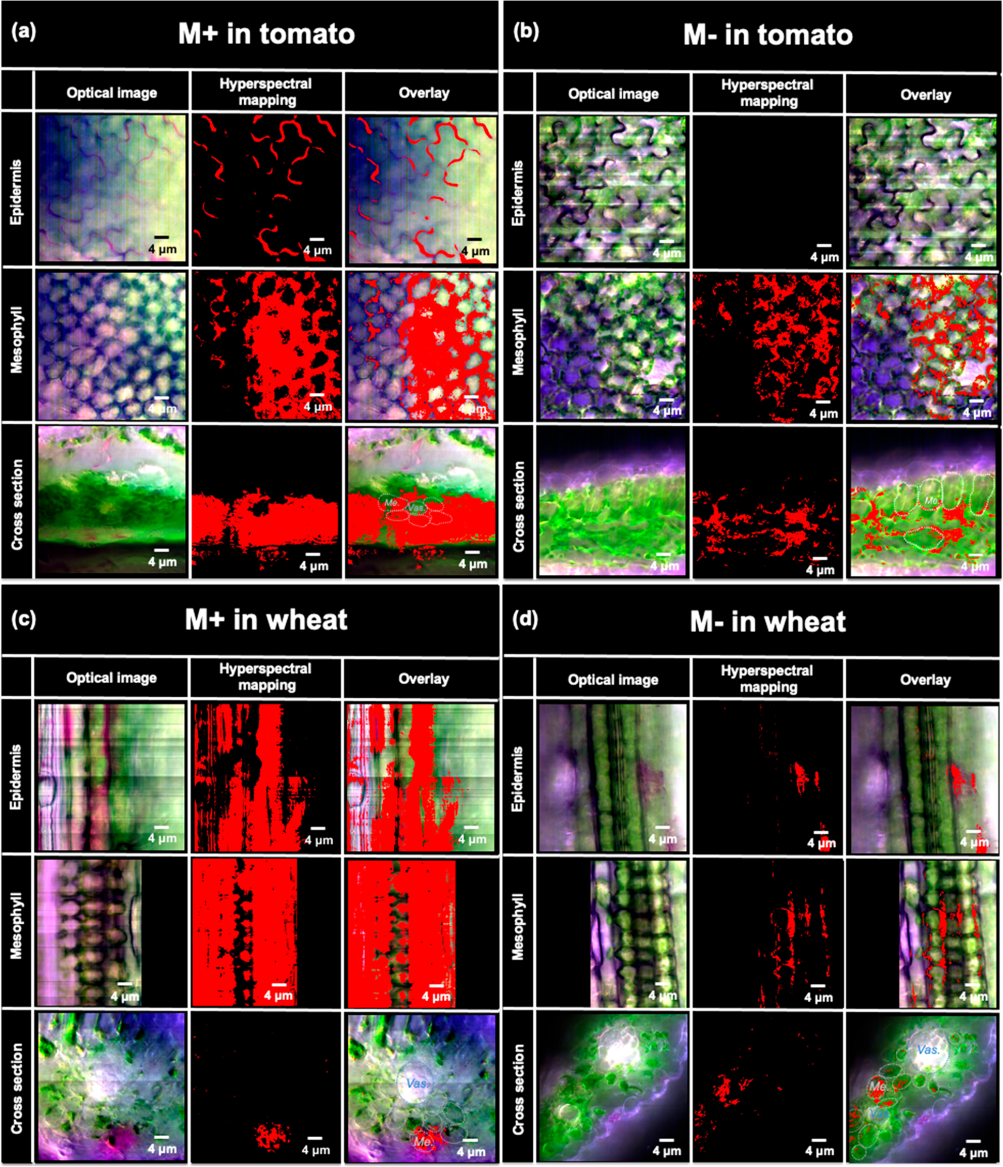
Interactions of RB loaded P[BiBEM-*g*-(PDMAEMA_50_-*b*-PNIPAm_50_)]_320_ bottlebrushes (M+) and CV loaded P[BiBEM-*g*-(PAA_50_-*b*-PNIPAm_50_)]_320_ bottlebrushes (M−) with tomato leaves (a,b) and wheat leaves (c,d) applied with Silwet L-77 surfactant (0.1 vol %) assessed by enhanced dark field hyperspectral imaging of leaf epidermis, mesophyll, and cross sections. Pixels containing the RB or CV loaded polymers are highlighted in red based on their hyperspectral signature (Figure S4). *Me.*: mesophyll cell; *Vas.*: vasculature bundle.

The observed differences in translocation to other parts of the plant may be a result of different amounts of uptake into the leaf or different transport behaviors in the leaf so the interactions of the M+ and M– nanocarriers with leaves were assessed using hyperspectral imaging. Images of the nanocarriers on the leaf epidermis, inside the mesophyll, and in the apoplastic space were acquired 24 h after foliar application of the M+ and M– nanocarriers (Figure 5). In dicot tomato plants, a significant fraction of cationic M+ was found on the epidermis layer of tomato leaf (Figure 5a). In contrast, there was no M– found on the tomato leaf epidermis (Figure 5b), suggesting that all of the anionic M– is transported through the epidermis and taken up into mesophyll or preferentially translocated across stomatal pores.^13^ A similar trend was found in monocot wheat plants, as more M+ was found on the wheat leaf epidermis than M– (Figure 5c,d). The positive charge may inhibit nanocarrier penetration through the plant leaf epidermis and cuticle through electrostatic interaction with the negatively charged biosurfaces that exist in the leaf epidermis,^37^ which potentially decrease their uptake into plants. This likely explains the lower overall transport of the cationic nanocarriers compared to the anionic nanocarriers observed in both plant species.

Even though more M+ was found on the epidermis than M–, both M+ and M– were found in leaf mesophyll of tomato and wheat plants (Figure 5a,b,c,d). This indicates that some amount of both anionic and cationic nanocarriers could penetrate the cuticle and epidermis and enter the leaf tissue. Once inside the leaf, the positively charged M+ had more uptake into both tomato and wheat mesophyll cells (Figure 5a,c) than the negatively charged M– (Figure 5b,d). This suggests that the positive charge promotes nanocarrier uptake into mesophyll cells after entering plant leaves. Previous studies have also shown that a positive charge can promote uptake of “hard” nanoparticles such as carbon dots and nanoceria by leaf mesophyll cells and chloroplasts of cotton and maize plants after foliar topical delivery.^25^ The current results also suggest that the charge of “soft” materials such as these polymer nanocarriers, or biopolymers that have been proposed as nanocarriers,^38,39^ may behave similarly to the charge from “hard” particles with respect to nanomaterial-mesophyll cell interactions despite differences in their stiffness. However, the influence of other properties such as the flexibility of polymer nanomaterials may affect their ability to penetrate through plant barriers such as the cell wall and cell membrane. The role of stiffness on plant-nanomaterial interactions and translocation remains to be explored.

The cationic nanocarrier with enhanced plant cell uptake could potentially be developed for DNA and siRNA delivery into plants for genetic engineering. Current genetic engineering tools such as agrobacterium only transfect a limited number of plant species.^16^ The cationic nanocarriers with efficient cell uptake in both monocot and dicot plants could overcome these limitations, with the potential to genetically modify crops with higher yields and more resistance to climate change.^16,40,41^

There were also differences in uptake of M+ between tomato and wheat plants. Images in tomato leaf cross sections show that the M+ were readily taken up by the mesophyll cells surrounding the vasculature bundle (Figure 5a). After foliar application and uptake across the cuticle and epidermis, nanoparticles can transport in leaf mesophyll through either apoplastic (in between cells) or symplastic (through cells) pathways.^12^ Due to the small size cutoff of the plasmodesmata (2–20 nm),^42^ nanocarrier symplastic transport may be more restricted than apoplastic transport, making the latter the primary pathway.^42^ While the cationic nanocarrier may favor uptake into plant cells, the greater uptake into mesophyll cells also causes them to be trapped in those cells. This lowers the mass of particles undergoing translocation through the apoplastic spaces and uptake into phloem companion cells and the plant vasculature, thereby lowering systemic translocation as was observed in this study. In contrast to tomato (dicot), M+ accumulates in fewer mesophyll cells in the wheat (monocot) cross section (Figure 5c), suggesting lower potential for loading into the plant vasculature. There was more evidence of the anionic M– spreading further to both symplastic and apoplastic spaces (Figure 5d), suggesting higher mobility of M– than M+ and greater potential for loading into the vasculature and translocation.

The differences in leaf surface structure, including stomatal density and cuticle thickness, may also determine nanocarrier uptake efficiency in wheat and tomato plants. The stomatal density on dicot plant leaves is around eight times higher than on monocot plant leaves,^25^ which may favor nanocarrier uptake through the stomata and subsequent translocation in tomato plants. The thicker cuticle on tomato leaf (∼0.3 μm)^43^ compared to wheat leaf (∼0.1 μm)^44^ could inhibit nanocarrier uptake, but the presence of a spreading agent like Silwet L-77 can disturb the thicker cuticle and promote uptake. The phyllosphere microorganisms in different plants could also potentially affect nanomaterial interaction with leaf surface and entrance into leaf mesophyll. This is largely unexplored in the literature and not addressed here, but effects from phyllosphere in different plants remain to be investigated.^45,46^

### Environmental Implications

These results suggest that different nanocarrier properties can be leveraged for the design of nanocarriers for more efficient and targeted agent delivery in crop plants to treat plant disease and deliver genetic elements. Current agrochemical application methods cannot effectively deliver active ingredients to some desired plant targets such as vasculature and root. Given the significant translocation into stem and root, an anionic nanocarrier carrying antimicrobial agents could be developed to target plant vascular disease such as HLB in citrus^47^ and wheat blast.^48,49^ This new treatment strategy could enable more effective plant vascular disease treatment, reduce crop loss, and promote sustainability of food production. The near 100% plant uptake and targeted agent delivery of the nanocarriers studied here can potentially avoid excess agrochemical application, which avoids agrochemical runoff and helps to alleviate environmental burdens of agriculture.

Plant species and leaf anatomy (monocot vs dicot) play an important role in nanocarrier translocation, and the general translocation trends observed in dicots cannot be directly applied to monocots. The total transport of nanocarrier in monocot wheat plants ranges from ∼3 to 9%, which is lower than the total transport in dicot tomato plants (8–25%). This is similar to previous studies exposing plant roots to 4 nm CeO_2_ nanoparticles, where the translocation efficiency of these particles through the vasculature of dicots (tomato and lettuce) was much higher than in monocots (maize and rice).^23^ The monocot plants in this study had a clear size cutoff for high aspect ratio nanocarriers, as both the 300 nm long H+ and H– could not transport in monocot wheat plants after foliar application. M– with 80 nm in length and a 10 nm diameter measured by AFM translocated in wheat with a reported 5–20 nm cell wall size cutoff,^50^ indicating that the smallest dimension may determine the translocation across this plant biosurface. The dicots did not have this limitation and appear to be more amenable to treatment with nanocarriers.

The synthetic polymer nanocarriers demonstrated high foliar uptake and good translocation and cell uptake. While the nanocarriers used here are composed of nontoxic polymers widely used for biomedical research,^31^ the effects of any commercially produced nanocarrier to long-term plant physiology and grain/fruit production would need to be taken into consideration in nanocarrier design. Moreover, limited biodegradability may lead to accumulation in the food chain.^51^ Future studies should therefore develop nanocarriers with a similar physical size and charge as those used here but using biodegradable polymers or natural polymers such as chitosan, cellulose or other polysaccharides, or silk or other peptides to address any potential concerns regarding biodegradability and biocompatibility.^52−55^ Here, we also only evaluated the short-term plant uptake and translocation behaviors of polymer nanocarriers. The long-term fate of nanocarriers needs to be explored, especially their potential transport into crop grains, fruits, and other edible parts of the plant.^11^ In addition, our current study demonstrates transport of model cargo molecules (metals and organic dye) in plants as these cargos enabled mapping of their distribution in plants, but future studies should use nanocarriers to deliver various agrochemicals such as nutrients, plant hormones, or other active ingredients and elucidate the benefits that they may provide over existing state of the art agrochemical delivery methods.^52,54,56,57^ It is important to note that loading the nanocarrier with a charged agrochemical can potentially change its charge; therefore, the properties of the loaded nanocarrier would likely determine the fate in the plant. Finally, the plant growth stage should also be considered for nanocarrier application. Cuticle properties can change with the growth stage, affecting leaf uptake.^58,59^ Application of nanocarriers to plants during fruiting can potentially lead to dietary exposure if phloem loaded nanocarriers are moved into the fruit. The influence of timing of nanocarrier application needs to be further studied to minimize these risks.

## Supporting Information Available

Detailed synthesis procedure for star polymers and bottlebrush polymers, AFM imaging, spectral library build up procedure, figures showing ^1^H NMR spectra of PDMAEMA-*b*-PNIPAm star polymers and P[BiBEM-*g*-(PDMAEMA-*b*-PNIPAm)] bottlebrushes, GPC trace and *M*_n_ of PDMAEMA star and bottlebrush polymers, DLS size, electrophoretic mobility and apparent zeta potential of polymer carriers, Gd loading in polymers, Gd leaching from polymers in simulated apoplastic fluid, uptake and transport of Gd-loaded positively charged polymer nanocarriers in wheat, uptake and transport of Gd-loaded positively charged polymer nanocarriers in tomato, and spectral library for hyperspectral mapping (PDF)
