# Supplementary material for: Charge, Aspect Ratio, and Plant Species Affect Uptake Efficiency and Translocation of Polymeric Agrochemical Nanocarriers
Source: Environ Sci Technol. 2023 May 25;57(22):8269–79. doi: 10.1021/acs.est.3c01154 (PMC10249409; doi:10.1021/acs.est.3c01154)
Supplement: Supplementary file 1 — es3c01154_si_001.pdf [file es3c01154_si_001.pdf]

Supporting information for:

Charge, Aspect Ratio and Plant Species Affects Uptake Efficiency and Translocation of Polymeric Agrochemical Nanocarriers

*Yilin Zhang [a,b], Michael R. Martinez [c], Hui Sun [g], Mingkang Sun [c], Rongguan Yin [c], Jiajun Yan [c], Benedetto Marelli [g], Juan Pablo Giraldo [f], Krzysztof Matyjaszewski [c], Robert D. Tilton [b,d,e] * and Gregory V. Lowry [a,b*]*

a. Department of Civil and Environmental Engineering, b. Center for Environmental Implications of Nano Technology (CEINT), c. Department of Chemistry, d. Department of Chemical Engineering e. Department of Biomedical Engineering, Carnegie Mellon University, Pittsburgh, Pennsylvania 15213, United States.

f. Department of Botany and Plant Sciences, University of California, Riverside, California 92521, United States.

g. Department of Civil and Environmental Engineering, Massachusetts Institute of Technology, Cambridge, Massachusetts 02139, United States.

* Corresponding authors

Phone: (412) 268-2948; fax: (412) 268-7813; email: glowry@cmu.edu (G.V. Lowry);

Phone: (412) 268-1159; fax: (412) 268-7139; email: tilton@cmu.edu (R.D. Tilton);

Number of Figures: 7
Number of Tables: 8
19 pages

Table of Contents

Synthesis of P[BiBEM-g-(PDMAEMA$_{50}$-b-PNIPAm$_{50}$)]$_{320}$ (M+) polymer bottlebrushes............3

Synthesis of P[BiBEM-g-(PDMAEMA$_{50}$-b-PNIPAm$_{50}$)]$_{1600}$ (H+) polymer bottlebrushes............3

Synthesis of polymer nanocarriers with PAA-b-PNIPAm side chains 4

Polymer characterization by atomic force microscopy, dynamic light scattering and ^{1}H NMR.....4

Spectral library building 6

Figure S2. ^{1}H NMR spectra of (a) PDMAEMA$_{50}$-b-PNIPAm$_{50}$ star polymer, (b) P[BiBEM-g-(PDMAEMA$_{50}$-b-PNIPAm$_{50}$)]$_{320}$ polymer bottlebrush and (c) P[BiBEM-g-(PDMAEMA$_{50}$-b-PNIPAm$_{50}$)]$_{1600}$ polymer bottlebrush in $CDCl_3$. 8

Figure S3. GPC traces of the polymer product of (A) 21-armed PDMAEMA$_{50}$ star polymer and (B) P[BiBEM-g-(PDMAEMA$_{50}$]$_{320}$ bottlebrush in DMF. 9

Table S1. Number average molecular weights (M_n) of PDMAEMA star polymers and P[BiBEM-g-PDMAEMA] polymer bottlebrushes in DMF measured by GPC-MALLS. 9

Table S2. Calculated Number Average Molecular Weight (M_n), electrophoretic mobility, apparent zeta potential and number average hydrodynamic diameter of polymer nanocarriers. ..10

Table S3. Aspect ratio of L+, M+ and H+ acquired from AFM height maps and analyzed by ImageJ. Aspect ratios were calculated by the ratio of nanocarrier length and width (diameter)...11

Table S4. Gd loading into different polymer carriers and free Gd detected outside of the dialysis bag...12

Table S5. Gd leaching out of different polymer carriers after dialysis against simulated apoplastic fluid.[8] 12

Table S6. Gd recovery (% of applied Gd) from plants post foliar application by ICP-MS. 13

Table S7. Calibration curve of Gd acquired by ICP-MS. 13

Table S8. Electrophoretic mobility, apparent zeta potential and number average hydrodynamic diameter of polymer nanocarriers loaded with Gd. 14

Figure S5. Control (without RB or CV labeled nanocarrier exposure) images of wheat and tomato plant mesophyll mapped by spectral library of (a) M+ in wheat, (b) M- in wheat, (c) M+ in tomato and (d) M- in tomato. Pixels containing the RB or CV labeled polymers are highlighted in red based on their hyperspectral signature (Figure S4). Lack of red highlights indicate polymer nanocarrier is not detected in the image. 16

Figure S6. Uptake and transport of Gd-loaded positively charged polymer nanocarriers in wheat plants after foliar application 17

Figure S7. Uptake and transport of Gd-loaded positively charged polymer nanocarriers in tomato plants after foliar application 18

Material synthesis

Synthesis of P[BiBEM-*g*-(PDMAEMA$_{50}$-*b*-PNIPAm$_{50}$)]$_{320}$ (M+) polymer bottlebrushes

PBiBEM$_{320}$ and PBiBEM$_{1600}$ polymer backbones are synthesized according to previous study.[1] The PDMAEMA block of bottlebrush was prepared by normal ATRP. Briefly, 0.05 g (1 equiv) of PBiBEM$_{320}$, 11.96 mL (160000 equiv) of DMAEMA, 3.8 mg (64 equiv) of $CuCl_2$, 0.039 mL (320 equiv) of HMTETA and 23.9 mL anisole were mixed in a sealed Schlenk flask equipped with a stir bar. The Schlenk flask was degassed by purging with N_2 for 60 min and the reaction was frozen in liquid nitrogen. The flask was opened briefly to add 14.1 mg (320 equiv) of CuCl powder to the frozen reaction. The flask was sealed again and purged with N_2 for 30 min before being allowed to warm to room temperature. The reaction was stopped at 10% conversion to yield P(BiBEM-*g*-PDMAEMA$_{50}$)$_{320}$ bottlebrush. The product was dialyzed against methanol for 3 cycles (MWCO=8000) to remove excess reagents. The molecular weight of polymer bottlebrushes was characterized with GPC-MALLS (**Figure S3b, Table S1**).

For PNIPAm chain extension, 0.12 g (1 equiv) of P(BiBEM-*g*-PDMAEMA$_{50}$)$_{320}$, 0.43 g (80000 equiv) of NIPAm, 0.68 mg (64 equiv) of $CuBr_2$, 0.0025 mL (192 equiv) of Me$_6$Tren and 19.1 mL of DMF were mixed in a sealed Schlenk flask equipped with a stir bar. The Schlenk flask was degassed by purging with N_2 for 60 min and the reaction was frozen in liquid nitrogen. The flask was opened briefly to add 0.058 g (0.68 cm^{-1}) of Cu^0 powder. The flask was sealed again and purged with N_2 for 30 min before being allowed to warm to room temperature. The reaction was stopped at 20% conversion to yield P[BiBEM-*g*-(PDMAEMA$_{50}$-*b*-PNIPAm$_{50}$)]$_{320}$ bottlebrush (SPBB(+)). The product was dialyzed against methanol for 3 cycles (MWCO=8000). The chemical composition of product was verified by ^{1}H NMR in $CDCl_3$ (**Figure S2b**).

Synthesis of P[BiBEM-*g*-(PDMAEMA$_{50}$-*b*-PNIPAm$_{50}$)]$_{1600}$ (H+) polymer bottlebrushes

Briefly, 0.04 g (1 equiv) of PBiBEM$_{1600}$, 10.98 mL (800000 equiv) of DMAEMA, 3.5 mg (320 equiv) of $CuCl_2$, 0.035 mL (1600 equiv) of HMTETA and 22 mL of anisole were mixed in a sealed Schlenk flask equipped with a stir bar. The reaction was degassed by N_2 and was frozen in liquid nitrogen. 0.013 g (1600 equiv) of CuCl was added to the reaction and the reaction was

degassed again by N_2 purging. Reaction was stopped at 10% conversion to yield P(BiBEM-*g*-PDMAEMA$_{50}$)$_{1600}$ bottlebrush.

For PNIPAm chain extension, 0.15 g (1 equiv) of PDMAEMA$_{50}$)$_{1600}$, 0.54 g (400000 equiv) of NIPAm, 0.86 mg (320 equiv) of $CuBr_2$, 0.0032 mL (960 equiv) of Me_6Tren and 20 mL of DMF were mixed in a Schlenk flask and degassed by purging with N_2. The reaction was frozen in liquid nitrogen and opened briefly to add 0.06 g (0.68 cm^{-1}) of Cu^0 powder. The flask was sealed again and purged with N_2 for 30 min before being allowed to warm to room temperature. The reaction was stopped at 20% conversion to yield P[BiBEM-*g*-(PDMAEMA$_{50}$-*b*-PNIPAm$_{50}$)]$_{1600}$ bottlebrush (LPBB(+)). The product was dialyzed against methanol for 3 cycles (MWCO=8000). The chemical composition of product was verified by ^{1}H NMR in $CDCl_3$ (**Figure S2c**).

Synthesis of polymer nanocarriers with PAA-*b*-PNIPAm side chains

The synthesis of PAA$_{50}$-*b*-PNIPAm$_{50}$ star polymers (L-), P[BiBEM-*g*-(PAA$_{50}$-*b*-PNIPAm$_{50}$)]$_{320}$ (M-) and P[BiBEM-*g*-(PAA$_{50}$-*b*-PNIPAm$_{50}$)]$_{1600}$ (H-) polymer bottlebrushes followed our previously published methods. [2]

Polymer characterization by atomic force microscopy, dynamic light scattering and ^{1}H NMR

Atomic force micrographs were obtained using a Cypher VRS AFM (Asylum Research). All samples were diluted to 10 mg L^{-1} for AFM imaging, in order to have an optimal density of features on the substrate. In a typical experiment, a 10 µL aliquot of diluted sample was dropped on freshly cleaved mica surface (ϕ = 10 mm, Ted Pella) and air dried before imaging. Images were acquired by tapping mode in air, at a scan rate of 4 - 8 Hz and a resolution of 256 × 256 pixels per image, using FS1500AuD (Asylum Research) probes.[3] Hydrodynamic diameter, electrophoretic mobility and apparent zeta potential of polymer nanocarriers were measured with 100 mg L^{-1} polymer concentration in 10 mM NaCl water solution at pH 6.5 using Malvern zetasizer nano zs. Apparent zeta potentials (ζ) were calculated from the mobility via the Smoluchowski model. The chemical composition of polymer nanocarriers were confirmed by ^{1}H NMR in $CDCl_3$ using a Bruker Advance 500 MHz NMR spectrometer.

Gd loading into nanocarriers for tracking their distribution in plants

Gadolinium-diethylenetriaminepentaacetic acid (Gd-DTPA) was loaded into the positively charged L+, M+ and H+ nanocarriers to enable tracking by inductively coupled plasma mass spectrometry (ICP-MS). Typically, 10 mg of cationic nanocarrier was dissolved into MilliQ water with 100 µL of 1 M HCl in an ice bath with sonication (iSonic P4800). The pH of the polymer solution was adjusted by adding either 0.1 M NaOH or 0.1 M HCl to reach pH=6.5. Then, 50 mg of Gd-DTPA was dissolved into the solution and mixed with a vortex mixer for 24 h to allow electrostatic binding between DMAEMA and Gd-DTPA. The mixture was dialyzed in 200 mL Milli-Q water (MWCO = 8000 Da) for seven cycles, until free Gd concentration in the dialysate contained less than 0.1% of Gd loaded in star polymers. Gd^{3+} was loaded into the anionic nanocarriers using a previously reported method.[4] The final Gd loading into cationic and anionic nanocarriers were measured by ICP-MS (Agilent 7700x). The stability of the loaded Gd against leaching in apoplastic fluid was also measured. In a typical procedure, 2 mL of Gd labeled nanocarrier was dialyzed against 100 mL of simulated apoplastic fluid.[7,10] The Gd loading in the different nanocarriers are shown in **Table S4**. Around 145-255 mg/g of Gd was loaded into polymer nanocarrier. Between 0.7-2.8% (most <1%) of the loaded Gd leached out of nanocarriers in simulated apoplastic fluid after 24 h (**Table S5**), suggesting that the Gd labeling in the nanocarriers is stable in plants and the Gd detected in different plant tissue is associated with polymers.[7] Gd loading in the anionic nanocarriers followed previously published procedures.[4] The size and charge of Gd loaded nanocarriers are summarized in **Table S8**.

Organic dye loading and polymer nanocarrier foliar application to image their leaf uptake pathway

The nanocarriers were labeled with organic dyes with strong light absorbance to track their distribution in plant leaves using hyperspectral imaging. The anionic Rose Bengal (RB) dye was used to label the cationic nanocarriers with PDMAEMA-*b*-PNIPAm side chains. Briefly, 10 mg of cationic nanocarrier was dissolved into MilliQ water with 100 µL of 1 M HCl in an ice bath with sonication (iSonic P4800). The pH of the polymer solution was adjusted to 6.5 using 0.1 M NaOH. Then, 5 mg of RB was added into the solution and mixed with a vortex mixer for 24 h. The mixture was dialyzed in 200 mL Milli-Q water (MWCO = 8000 Da) for seven cycles, until RB is undetectable by UV-Vis spectrophotometry (Agilent Cary 4000). Crystal violet (CV) was used to

label the anionic polymer nanocarriers with PAA-*b*-PNIPAm side chains using a previously reported method.

Spectral library building

The loaded polymer nanocarrier spectral libraries were built based on images of the polymer nanocarriers on leaves, using the following steps according to previous literature: [4,5]

(i) Spectral data reduction: The images were transformed into minimum noise fraction (MNF) images, where a noise covalence matrix is used to decorrelate and rescale the noise in the data (algorithm adapted from Green et al., 1988).[6] Coherent MNF images (containing spectral information with minimal noise) can then be separated from the noise-dominated ones.

(ii) Endmember identification and pre-library building: A pixel purity index serves to find the most spectrally pure pixels in the images. This PPI is used as input parameter in an n-dimensional visualizer (n is the number of reflectance spectra or pixels), in which an n-dimensional vector represents each spectrum. This visualization allows identifying and grouping the purest pixels, i.e. the most extreme spectral responses (here termed endmembers). The endmembers constitute the pre-library. The pre-library can still contain some spectra of materials other than the loaded star polymer. Endmembers Library building: The hyperspectral libraries were filtered using the "spectral filter" option of the software ENVI 5.2. During that step all of the hyperspectral signal coming from a control leaf were filtered out. This was done using a spectral angular mapping algorithm (SAM). The SAM algorithm provides a measure for the similarity between 2 spectrums (here between the one in the pre-library and the ones on the pixels of a control image) calculating the angle between the two spectra (again treated as n-dimensional vectors). The angle for SAM processing was set as the lowest level allowing the identification of endmembers in their own pictures (i.e. 0.085 rad). Vectors with angles $\leq$0.085 rad were considered as similar. The spectra (vectors) in the pre-library that matched pixels on hyperspectral images of the negative control leaves were considered as false positives and removed from the pre-library. The remaining spectra built the final hyperspectral library (i.e. a hyperspectral of loaded polymer nanocarrier specific signature). These libraries are show in **Figure S6**.

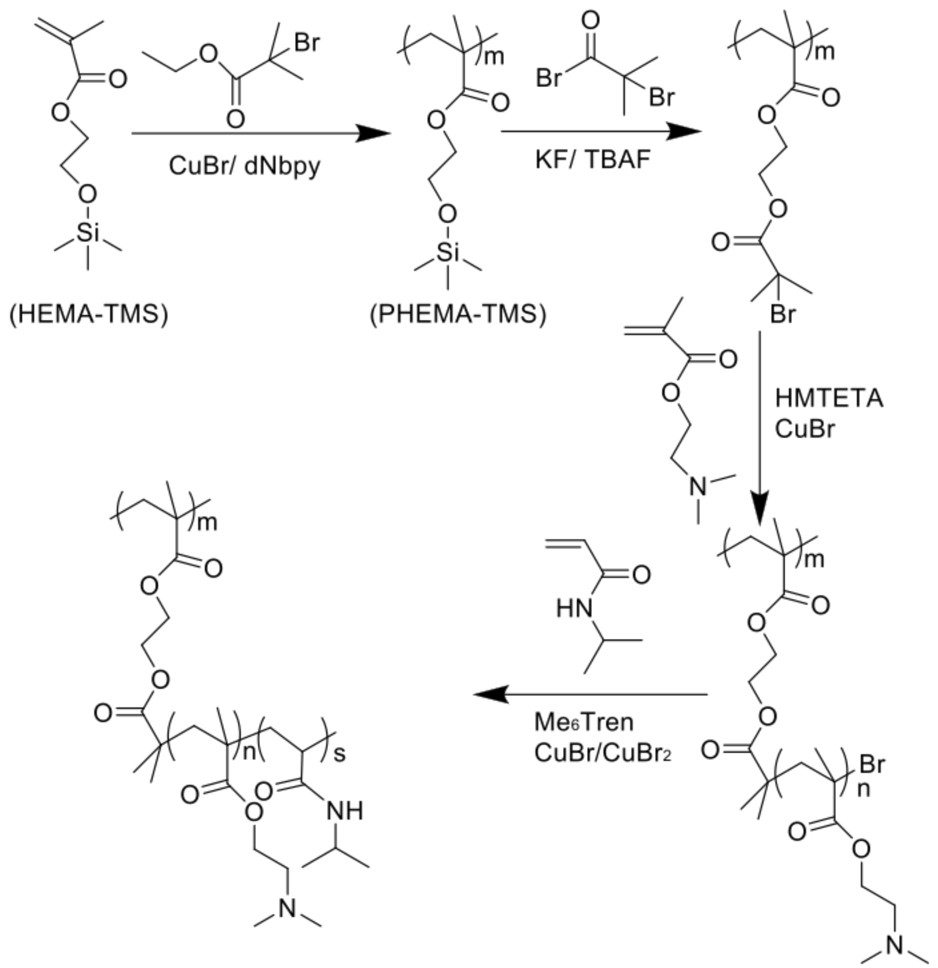

Figure S1. Synthesis procedure of the cationic high aspect ratio P[BiBEM-*g*-(PDMAEMA-*b*-PNIPAm)] polymer bottlebrushes.

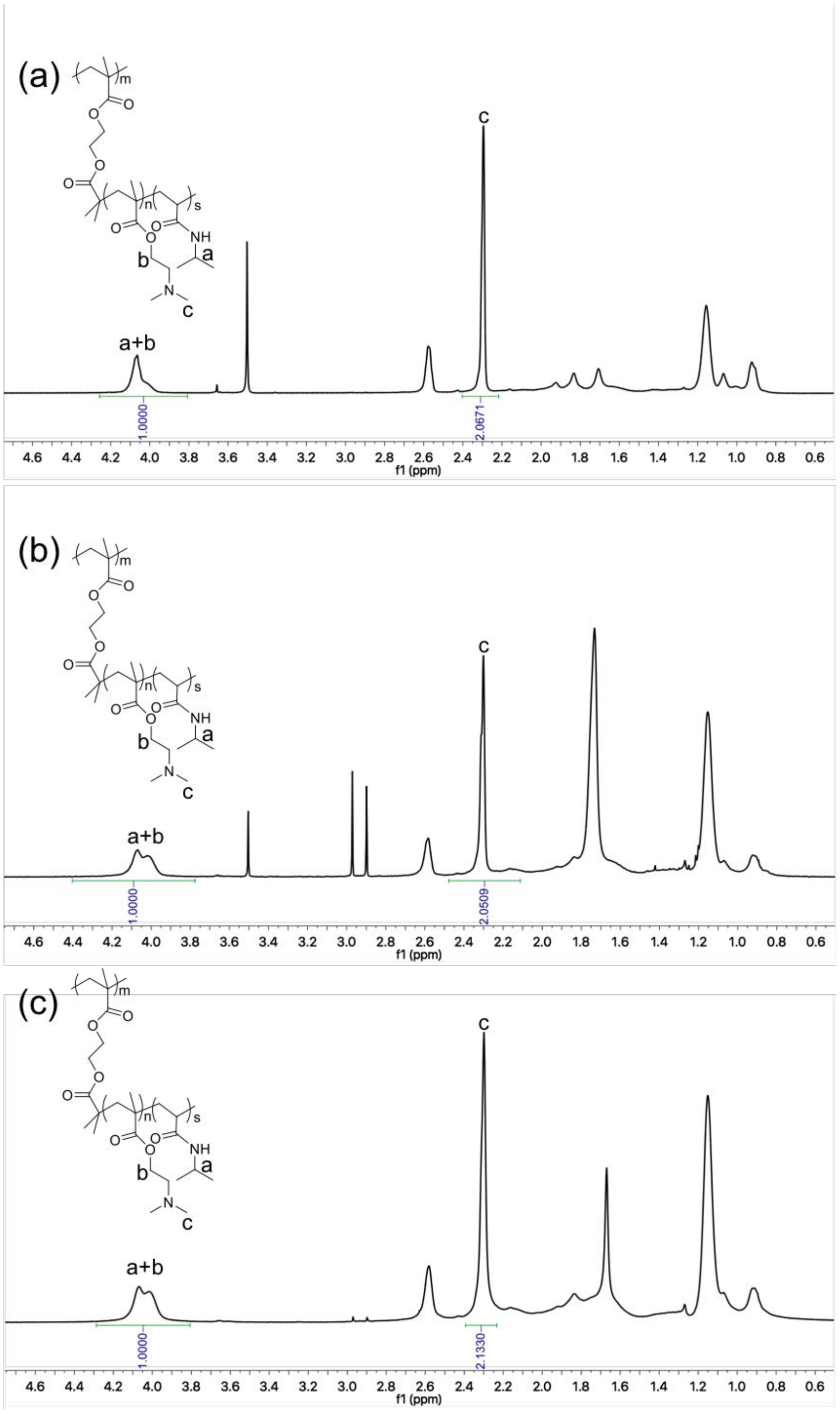

Figure S2. ^{1}H NMR spectra of (a) $PDMAEMA_{50}$-*b*-$PNIPAm_{50}$ star polymer, (b) P[BiBEM-*g*-($PDMAEMA_{50}$-*b*-$PNIPAm_{50}$)$]_{320}$ polymer bottlebrush and (c) P[BiBEM-*g*-($PDMAEMA_{50}$-*b*-$PNIPAm_{50}$)$]_{1600}$ polymer bottlebrush in $CDCl_3$.

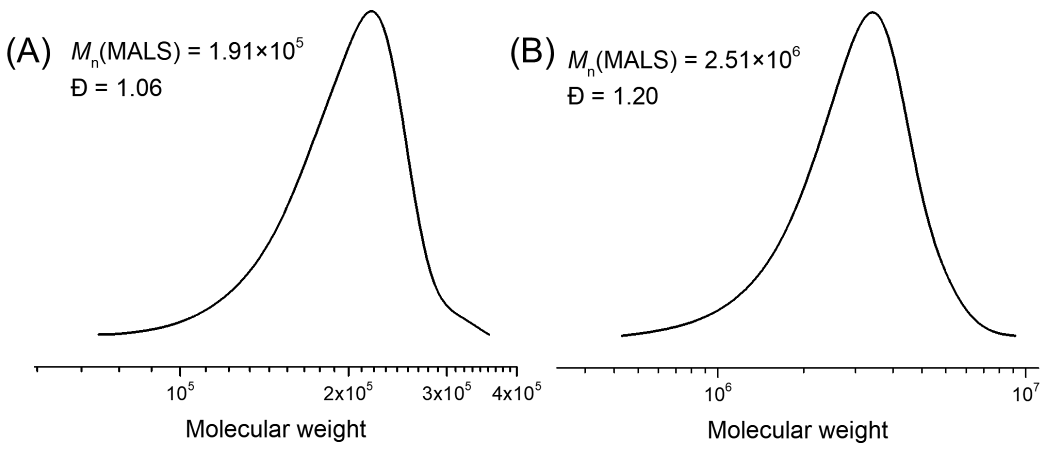

Figure S3. GPC traces of the polymer product of (A) 21-armed $PDMAEMA_{50}$ star polymer and (B) P[BiBEM-*g*-$(PDMAEMA_{50}]_{320}$ bottlebrush in DMF.

Table S1. Number average molecular weights (M_n) of PDMAEMA star polymers and P[BiBEM-*g*-PDMAEMA] polymer bottlebrushes in DMF measured by GPC-MALLS.

Sample	$M_{n, MALLS}$	*Đ*
**$PDMAEMA_{50}$ star polymer**	1.91×10^5	1.20
**P[BiBEM-*g*-$(PDMAEMA_{50}]_{320}$ bottlebrush**	2.51×10^6	1.05

Conditions: Gel permeation chromatography (GPC) was measured via a PSS GPC system with an Agilent 1260 Infinity II isocratic pump, a column set containing 3 PSS GRAM analytical columns (pore sizes: 3000 Å, 3000 Å, 100 Å), an Agilent 1260 Infinity II refractive index (RI) detector, and a PSS SLD2020 multi-angle light scattering (MALS) detector. The column set was kept in a column oven thermostatted at 50 ºC. DMF containing 0.05 M LiBr was used as the eluent. Analysis of polymer signals: PSS WinGPC software (build 9666) was used for molecular weight analysis. RI detector was used as the concentration detector. MALS-based molecular weights were calculated using "concentration × dn/dc" method. The dn/dc value of 0.056 was obtained from previous study.[7]

Table S2. Calculated Number Average Molecular Weight (M_n), electrophoretic mobility, apparent zeta potential and number average hydrodynamic diameter of polymer nanocarriers.

**Sample**	**M_n[a] (g mol^{-1})**	**Electrophoretic mobility (μm cm V^{-1} s^{-1})[b]**	**ζ (mV)[c]**	**D_h[d](nm)**
**$PDMAEMA_{50}$-*b*-$PNIPAm_{50}$ star (L+)**	2.84×10^5	0.94±0.58	11.9±7.5	14.3±3.5
**P[BiBEM-*g*-($PDMAEMA_{50}$-*b*-$PNIPAm_{50}$)]$_{320}$ bottlebrush (M+)**	4.32×10^6	1.3±0.03	16.5±0.4	40.9±2.6
**P[BiBEM-*g*-($PDMAEMA_{50}$-*b*-$PNIPAm_{50}$)]$_{1600}$ bottlebrush (H+)**	2.20×10^7	1.5±0.06	19.2±0.7	158.4±3.8
**PAA_{50}-*b*-$PNIPAm_{50}$ star (L-)**	1.76×10^5	-3.8±0.94	-48.5±12	13.7±4
**P[BiBEM-*g*-(PAA_{50}-*b*-$PNIPAm_{50}$)]$_{320}$ bottlebrush (M-)**	2.96×10^6	-1.3±0.04	-16.5±0.6	39.5±5.4
**P[BiBEM-*g*-(PAA_{50}-*b*-$PNIPAm_{50}$)]$_{1600}$ bottlebrush (H-)**	1.53×10^7	-1.3±0.05	-15.4±0.6	105.5±5.3

[a] Number average molecular weight of polymer nanocarriers calculated by their conversion according to ^{1}H NMR. [b] Electrophoretic mobility of polymers measured in 10 mM NaCl at pH 6.5 with 100 mg L^{-1} polymer concentration. [c] Zeta potential was converted from electrophoretic mobility based on Smoluchowski equation. [d] Number average hydrodynamic diameter measured by DLS at 100 mg L^{-1} Gd-loaded polymer concentration at pH 6.5 in 10 mM NaCl.

Table S3. Aspect ratio of L+, M+ and H+ acquired from AFM height maps and analyzed by ImageJ. Aspect ratios were calculated by the ratio of nanocarrier length and width (diameter).

**L+**		**Aspect raio: 1.07±0.27**			
**length**	**avg**	**stdev**	**width**	**avg**	**stdev**
13.04			10.9		
11.34			13.9		
14.41			10.8		
7.17			8.3		
6.95			5.23		
7.01			11.4		
10.8			7.77		
6.97	9.94066667	2.22569882	8.96	9.33466667	2.2996331
9.65			8.26		
9.57			10.5		
11.5			11.7		
10			6.52		
10			6.57		
10.2			9.56		
10.5			9.65		
**M+**		**Aspect raio: 8.21±2.53**			
**length**	**avg**	**stdev**	**width**	**avg**	**stdev**
70.27			7.05		
60.44			10.39		
73.68			12.09		
82.69			11.47		
91.85			9.97		
88.25			13.35		
75.1			8.54		
89.04	80.244	12.3382522	6.17	9.78266667	2.27055206
99.56			11.46		
62.9			10.4		
70.67			5.41		
87.31			8.72		
92.37			12.09		
92.51			9.67		
67.02			9.96		
**H+**		**Aspect raio: 28.54±9.77**			
**length**	**avg**	**stdev**	**width**	**avg**	**stdev**
142.8			18.22		
245.45			12.76		
332.83			8.85		
268.11			8.84		
295.02			12.77		
525.32			9.02		
232.13			7.91		
250.59	299.715333	84.1147145	7.92	10.5006667	2.82856215
270.33			11.87		
290.14			11.19		
358.15			8.85		
324.16			10.62		
295.68			7.29		
285.84			9.02		
379.18			12.38		

Table S4. Gd loading into different polymer carriers and free Gd detected outside of the dialysis bag.

Sample name	Gd loading in polymer, mg Gd g^{-1} polymers	Free Gd out of polymers (mg L^{-1})	Free Gd release (%)
**$PDMAEMA_{50}$-*b*-$PNIPAm_{50}$ star (L+)**	255	0.053	**0.02**
**P[BiBEM-*g*-($PDMAEMA_{50}$-*b*-$PNIPAm_{50}$)]$_{320}$ bottlebrush (M+)**	153	0.023	0.015
**P[BiBEM-*g*-($PDMAEMA_{50}$-*b*-$PNIPAm_{50}$)]$_{1600}$ bottlebrush (H+)**	197	0.016	0.008
**PAA_{50}-*b*-$PNIPAm_{50}$ star (L-)**	182.5	0.008	0.004
**P[BiBEM-*g*-(PAA_{50}-*b*-$PNIPAm_{50}$)]$_{320}$ bottlebrush (M-)**	174.7	0.011	0.006
**P[BiBEM-*g*-(PAA_{50}-*b*-$PNIPAm_{50}$)]$_{1600}$ bottlebrush (H-)**	144.7	0.003	0.002

Table S5. Gd leaching out of different polymer carriers after dialysis against simulated apoplastic fluid.[8]

Sample name	Gd leaching out (mg L^{-1})	Fraction of Gd leached out (%)
**$PDMAEMA_{50}$-*b*-$PNIPAm_{50}$ star (L+)**	0.034	0.7
**P[BiBEM-*g*-($PDMAEMA_{50}$-*b*-$PNIPAm_{50}$)]$_{320}$ bottlebrush (M+)**	0.023	0.8
**P[BiBEM-*g*-($PDMAEMA_{50}$-*b*-$PNIPAm_{50}$)]$_{1600}$ bottlebrush (H+)**	0.026	0.7
**PAA_{50}-*b*-$PNIPAm_{50}$ star (L-)**	0.087	2.4
**P[BiBEM-*g*-(PAA_{50}-*b*-$PNIPAm_{50}$)]$_{320}$ bottlebrush (M-)**	0.082	2.8
**P[BiBEM-*g*-(PAA_{50}-*b*-$PNIPAm_{50}$)]$_{1600}$ bottlebrush (H-)**	0.022	0.8

Table S6. Gd recovery (% of applied Gd) from plants post foliar application by ICP-MS.

Sample name	Gd recovery from wheat	Gd recovery from tomato
**PDMAEMA$_{50}$-*b*-PNIPAm$_{50}$ star (L+)**	104.1 %	103.3 %
**P[BiBEM-*g*-(PDMAEMA$_{50}$-*b*-PNIPAm$_{50}$)]$_{320}$ bottlebrush (M+)**	77.1 %	83.9 %
**P[BiBEM-*g*-(PDMAEMA$_{50}$-*b*-PNIPAm$_{50}$)]$_{1600}$ bottlebrush (H+)**	85.0 %	107.1 %
**PAA$_{50}$-*b*-PNIPAm$_{50}$ star (L-)**	71.3 %	83.6 %
**P[BiBEM-*g*-(PAA$_{50}$-*b*-PNIPAm$_{50}$)]$_{320}$ bottlebrush (M-)**	77.0 %	93.3%
**P[BiBEM-*g*-(PAA$_{50}$-*b*-PNIPAm$_{50}$)]$_{1600}$ bottlebrush (H-)**	106.2 %	69.7%

Table S7. Calibration curve of Gd acquired by ICP-MS.

Sample Name	Count Per Second	Measured Concentration
**0 ppb**	133.354	0
**0.5 ppb**	297.83	0.4815
**1 ppb**	488.972	1.0411
**5 ppb**	1880.382	5.1145
**25 ppb**	8819.06	25.428
**100 ppb**	35193.922	102.6416
**250 ppb**	86180.354	251.9071

Table S8. Electrophoretic mobility, apparent zeta potential and number average hydrodynamic diameter of polymer nanocarriers loaded with Gd.

Sample	Electrophoretic mobility (µm cm V^{-1} s^{-1})[a]	ζ (mV)[b]	D_h[c](nm)
**Gd loaded $PDMAEMA_{50}$-*b*-$PNIPAm_{50}$ star (L+)**	0.49±0.04	6.1±0.6	15.9±4.6
**Gd loaded P[BiBEM-*g*-($PDMAEMA_{50}$-*b*-$PNIPAm_{50}$)]$_{320}$ bottlebrush (M+)**	0.57±0.2	7.3±2.6	41.7±2.9
**Gd loaded P[BiBEM-*g*-($PDMAEMA_{50}$-*b*-$PNIPAm_{50}$)]$_{1600}$ bottlebrush (H+)**	0.52±0.04	6.5±0.4	144.6±9.8
**Gd loaded PAA_{50}-*b*-$PNIPAm_{50}$ star (L-)**	-0.53±0.15	-6.8±1.9	25.1±6.9
**Gd loaded P[BiBEM-*g*-(PAA_{50}-*b*-$PNIPAm_{50}$)]$_{320}$ bottlebrush (M-)**	-0.48±0.04	-6.1±0.5	52.2±14.4
**Gd loaded P[BiBEM-*g*-(PAA_{50}-*b*-$PNIPAm_{50}$)]$_{1600}$ bottlebrush (H-)**	-0.42±0.03	-5.3±0.3	123.8±7.1

[a] Electrophoretic mobility of polymers measured in 10 mM NaCl at pH 6.5 with 100 mg L^{-1} polymer concentration. [b] Zeta potential was converted from electrophoretic mobility based on Smoluchowski equation. [c] Number average hydrodynamic diameter measured by DLS at 100 mg L^{-1} Gd-loaded polymer concentration at pH 6.5 in 10 mM NaCl.

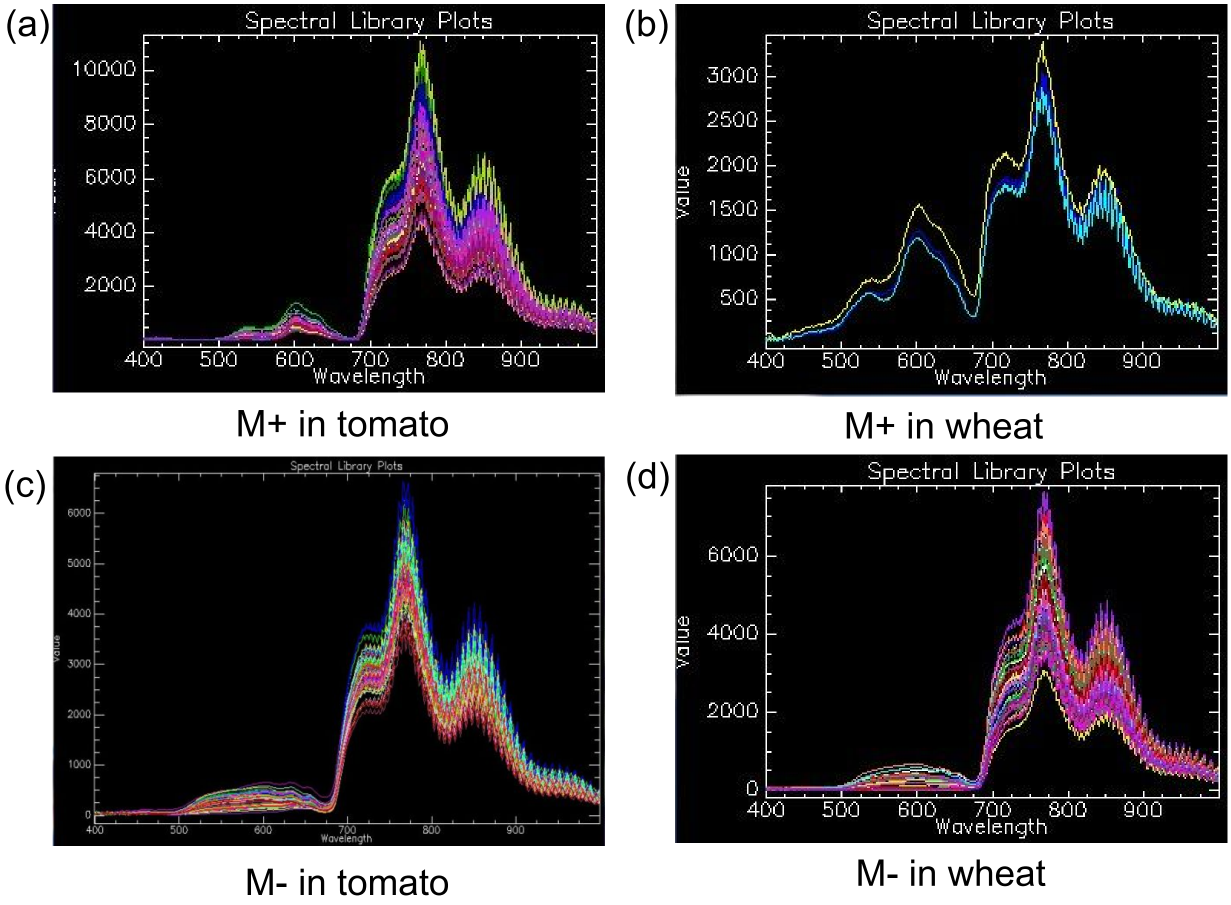

Figure S4. Spectral library of RB loaded (a) M+ in tomato, (b) M+ in wheat, (c) CV loaded M- in tomato, (d) M- in wheat.

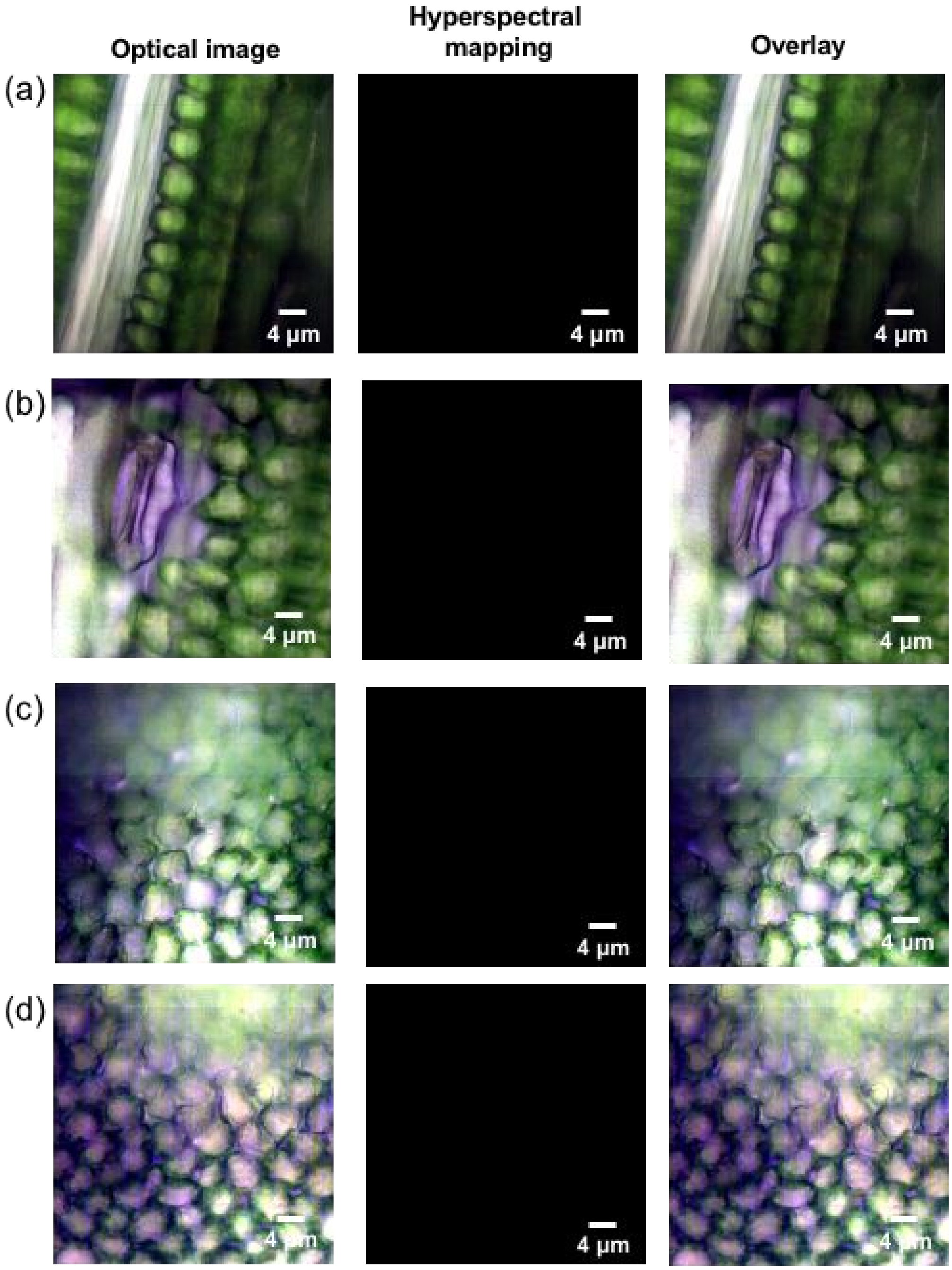

Figure S5. Control (without RB or CV labeled nanocarrier exposure) images of wheat and tomato plant mesophyll mapped by spectral library of (a) M+ in wheat, (b) M- in wheat, (c) M+ in tomato and (d) M- in tomato. Pixels containing the RB or CV labeled polymers are highlighted in red based on their hyperspectral signature (**Figure S4**). Lack of red highlights indicate polymer nanocarrier is not detected in the image.

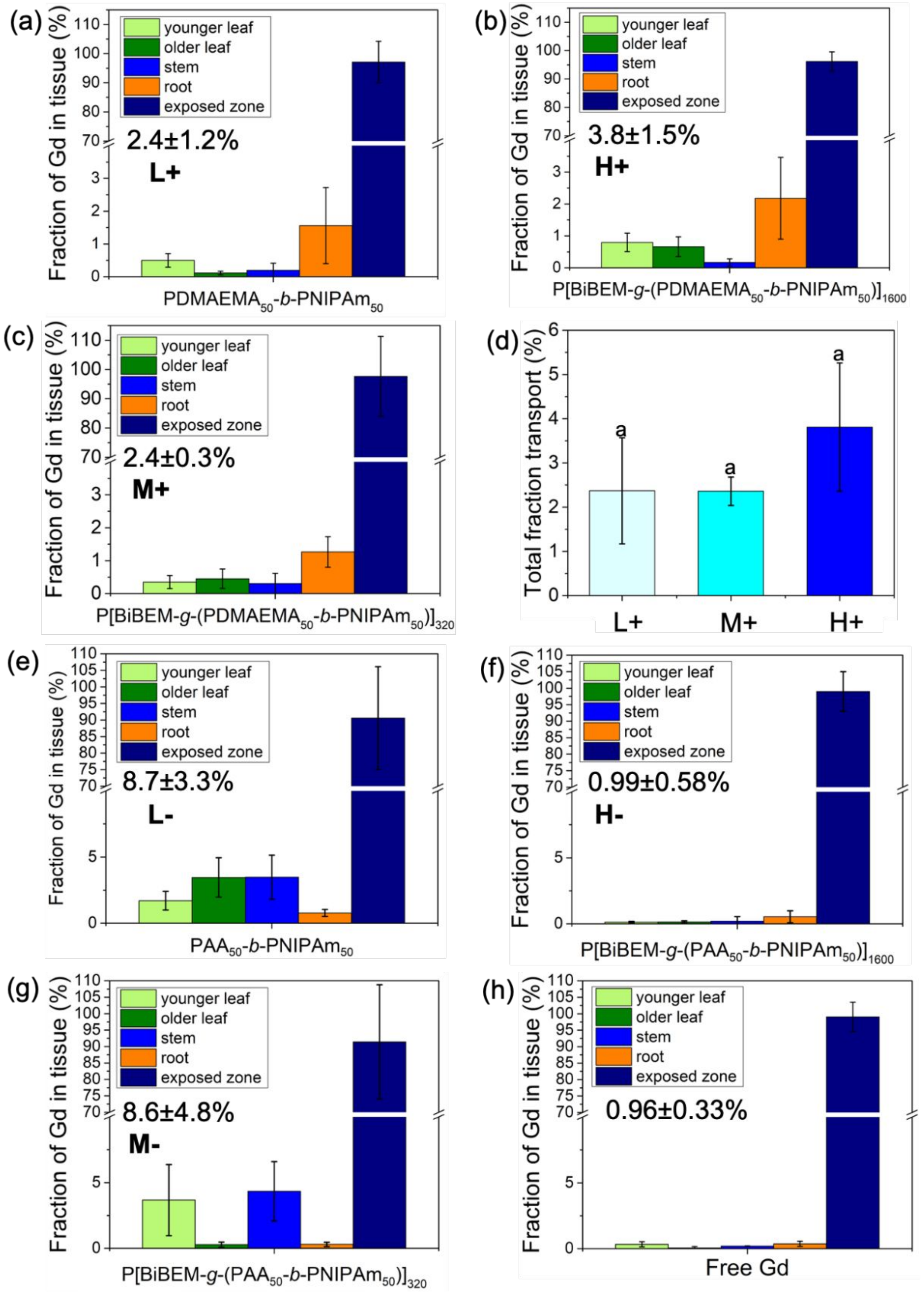

Figure S6. Uptake and transport of Gd-loaded positively charged polymer nanocarriers in wheat plants after foliar application of 20 µl of 1 g L^{-1} polymer with 0.1 v/v% Silwet L-77 for (a) $PDMAEMA_{50}$-*b*-$PNIPAm_{50}$ star polymers (L+), (b) P[BiBEM-*g*-($PDMAEMA_{50}$-*b*-$PNIPAm_{50}$)]$_{1600}$ bottlebrush (H+), (c) P[BiBEM-*g*-($PDMAEMA_{50}$-*b*-$PNIPAm_{50}$)]$_{320}$ bottlebrush

(M+) and (d) total fraction of Gd loaded polymer nanocarriers transported out of exposed leaf to other plant organs. Uptake and transport of Gd-loaded positively charged polymer nanocarriers in wheat for (e) PAA_{50}-*b*-$PNIPAm_{50}$ star polymers (L-), (f) P[BiBEM-*g*-(PAA_{50}-*b*-$PNIPAm_{50}$)]$_{1600}$ bottlebrush (H-), (g) P[BiBEM-*g*-(PAA_{50}-*b*-$PNIPAm_{50}$)]$_{1600}$ bottlebrush (M-) and (h) free Gd (200 mg L^{-1} Gd in Gd-DTPA) in wheat plants.

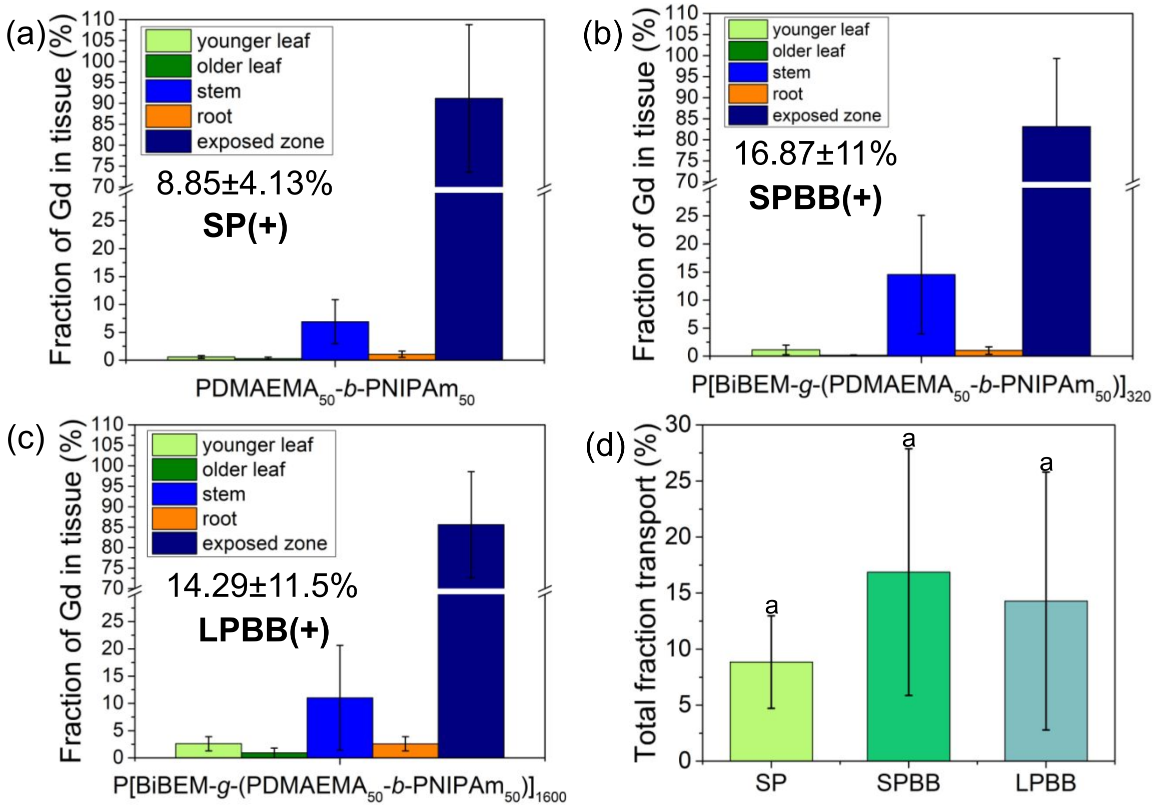

Figure S7. Uptake and transport of Gd-loaded positively charged polymer nanocarriers in tomato plants after foliar application of 20 µl of 1 g L^{-1} polymer with 0.1 v/v% Silwet L-77 for (a) $PDMAEMA_{50}$-*b*-$PNIPAm_{50}$ star polymers (L+), (b) P[BiBEM-*g*-($PDMAEMA_{50}$-*b*-$PNIPAm_{50}$)]$_{320}$ bottlebrush (M+) and (c) P[BiBEM-*g*-($PDMAEMA_{50}$-*b*-$PNIPAm_{50}$)]$_{1600}$ bottlebrush (H+).

References

(1) Zaborniak, I.; Chmielarz, P.; Martinez, M. R.; Wolski, K.; Wang, Z.; Matyjaszewski, K. Synthesis of High Molecular Weight Poly(n-Butyl Acrylate) Macromolecules via SeATRP: From Polymer Stars to Molecular Bottlebrushes. *Eur. Polym. J.* **2020**, *126*, 109566.

(2) Zhang, Y.; Yan, J.; Avellan, A.; Gao, X.; Matyjaszewski, K.; Tilton, R. D.; Lowry, G. V. Temperature- And PH-Responsive Star Polymers as Nanocarriers with Potential for in Vivo Agrochemical Delivery. *ACS Nano* **2020**, *14* (9), 10954–10965.

(3) Sun, H.; Marelli, B. Polypeptide Templating for Designer Hierarchical Materials. *Nat. Commun. 2020 111* **2020**, *11* (1), 1–13.

(4) Badireddy, A. R.; Wiesner, M. R.; Liu, J. Detection, Characterization, and Abundance of Engineered Nanoparticles in Complex Waters by Hyperspectral Imagery with Enhanced Darkfield Microscopy. **2012**.

(5) Avellan, A.; Schwab, F.; Masion, A.; Chaurand, P.; Borschneck, D.; Vidal, V.; Rose, J.; Santaella, C.; Levard, C. Nanoparticle Uptake in Plants: Gold Nanomaterial Localized in Roots of *Arabidopsis Thaliana* by X-Ray Computed Nanotomography and Hyperspectral Imaging. *Environ. Sci. Technol.* **2017**, *51* (15), 8682–8691.

(6) Green, A. A.; Berman, M.; Switzer, P.; Craig, M. D. A Transformation for Ordering Multispectral Data in Terms of Image Quality with Implications for Noise Removal. *IEEE Trans. Geosci. Remote Sens.* **1988**, *26* (1), 65–74.

(7) Methacrylate, N.; Situ, I. Antimicrobial Activity of Hybrid Nanomaterials Based on Star and Linear Polymers Of. *Materials (Basel).* **2020**, *13*, 3037.

(8) Zhang, Y.; Fu, L.; Li, S.; Yan, J.; Sun, M.; Pablo Giraldo, J.; Matyjaszewski, K.; D. Tilton, R.; V. Lowry, G. Star Polymer Size, Charge Content, and Hydrophobicity Affect Their Leaf Uptake and Translocation in Plants. *Environ. Sci. & Technol.* **2021**, *55* (15), 10758–10768.
